# Association between concurrence of multiple risk factors and under-5 mortality: a pooled analysis of data from Demographic and Health Survey in 61 low-and-middle-income countries

**DOI:** 10.1016/j.eclinm.2024.102583

**Published:** 2024-04-05

**Authors:** Yuhao Kong, Shaoru Chen, Ning Ma, Zekun Chen, Peter Karoli, John Lapah Niyi, Pengyang Fan, Günther Fink, Xiaoxiao Jiang Kwete, Fernando C. Wehrmeister, Feng Cheng, Dongqing Wang, Melkamu Aderajew Zemene, Samwel Maina Gatimu, Nuruzzaman Khan, Ashfikur Rahman, Lelisa Fekadu, Gebretsadik Shibre, Lhuri Dwianti Rahmartani, Justice Moses K. Aheto, Pascal Geldsetzer, Zhihui Li

**Affiliations:** a Vanke School of Public Health, Tsinghua University, Beijing, China; b National Institute for Medical Research, Dar es salaam, Tanzania; c Ghana Health Service, Gushegu Municipal Health Directorate, Gushegu, Ghana; d Swiss TPH and University of Basel, Basel, Switzerland; e Global Health Research and Consulting, Yaozhi, Yangzhou, China; f Department of Epidemiology, Universidade Federal de Pelotas, Pelotas, Brazil; g Institute for Global Public Health, Rady Faculty of Health Sciences, University of Manitoba, Winnipeg, Canada; h Institute for Healthy China, Tsinghua University, 100084, Beijing, China; i Department of Global and Community Health, College of Public Health, George Mason University, Fairfax, VA, USA, 22030; j Department of Public Health, College of Health sciences, Debre Tabor University, Debre Tabor, Ethiopia; k Diabetes Foot Foundation of Kenya, Kenya; l Centre for Women's Health Research, Faculty of Health and Medicine, University of Newcastle, Australia; m Development Studies Discipline, Social Science School, Khulna University, Bangladesh; n Health Economist, Health Economics and Financing Program, Africa CDC, Addis Ababa, Ethiopia; o Department of Reproductive, Family, and Population Health, School of Public Health, Addis Ababa University, Addis Ababa, Ethiopia; p Faculty of Public Health, Department of Epidemiology, Universitas Indonesia, Depok, Jawa Barat, Indonesia; q Department of Biostatistics, School of Public Health, University of Ghana, Ghana; r WorldPop, School of Geography and Environmental Science, University of Southampton, United Kingdom; s Division of Primary Care and Population Health, Department of Medicine, Stanford University School of Medicine, Stanford, CA, USA; t Chan Zuckerberg Biohub – San Francisco, CA, USA

**Keywords:** Under-5 mortality, Concurrence of multiple risk factors, Low-and middle-income countries

## Abstract

**Background:**

Exposure to multiple risk factors is prevalent in low-and middle-income countries (LMICs), challenging one-directional strategies to address preventable under-5 mortality (U5M). This study aims to assess the associations between concurrence of multiple risk factors and U5M in LMICs.

**Methods:**

We extracted data from the Demographic and Health Surveys conducted between 2010 and 2021 across 61 LMICs. Our primary outcome was U5M, defined as deaths from birth to 59 months. Binary logistic regression model was applied to ascertain the association between U5M and a total of 20 critical risk factors. Upon identifying the risk factors demonstrating the strongest associations, we investigated the simultaneous presence of multiple risk factors in each individual and assessed their combined effects on U5M with logistic regression models.

**Findings:**

Of the 604,372 under-5 children, 18,166 (3.0%) died at the time of the survey. Unsatisfied family planning needs was the strongest risk factor for U5M (odds ratio [OR]: 2.0, 95% confidence interval [CI]: 1.9–2.1), followed by short birth interval (<18 months; OR: 2.0, 95% CI: 1.9–2.1), small birth size (OR: 2.0, 95% CI: 1.8–2.1), never breastfed or delayed breastfeeding (OR: 2.0, 95% CI: 1.9–2.0), and low maternal education (OR: 1.6, 95% CI: 1.4–1.8). 66.7% (66.6%–66.8%) of the children had 2 or more leading risk factors simultaneously. Simultaneous presence of multiple leading risk factors was significantly associated with elevated risk of U5M and children presenting with all 5 leading risk factors exhibited an exceedingly high risk of U5M (OR: 5.2, 95% CI: 4.3–6.3); a dose–response relationship between the number of risk factors and U5M was also observed–with the increment of numbers of leading risk factors, the U5M showed an increasing trend (*p-trend* < 0.001).

**Interpretation:**

Exposure to multiple risk factors is very common in LMICs and underscores the necessity of developing multisectoral and integrated approaches to accelerate progress in reducing U5M in line with the SDG 3.2.

**Funding:**

This research is funded by Research Fund, Vanke School of Public Health, Tsinghua University.

**Research in context**

**Evidence before this study**

The global burden of under-5 mortality (U5M) remains substantial and is mainly concentrated on low-and-middle-income countries (LMICs). We conducted a thorough search of Google Scholar, Web of Science, and PubMed. Our search included various combinations of the following terms: (“under-5 child mortality” [All Fields] OR “under-5 mortality” [All Fields] OR “under-5 child death” [All Fields] OR “child health” [All Fields]) AND (“risk factor” [All Fields] OR “causes” [All Fields] OR “intervention” [All Fields] OR “prevention” [All Fields] OR “identification” [All Fields]) AND “low-and middle-income countries [All Fields].” We did not impose any restrictions on publication date or language, and our last search was conducted on July 3rd, 2023. The available literature reported the role of household wealth, maternal education, place of residence, number of births and number of total children ever born, exposure to indoor pollution, and lack of access to improved sanitation, contraceptive use and intent, sex and weight of child at birth, type of toilet facility, and breastfeeding as important contributors to U5M in LMICs. While numerous studies have identified these risk factors and causes of U5M, most of them have overlooked the crucial fact that the majority of children from LMICs suffer from multiple risk factors simultaneously.

**Added value of this study**

The significance of our study lies in its meticulous examination of numerous risk factors on child mortality, and we also attempt to provide a comprehensive understanding of how the combination and interplay of these risk factors impact child mortality. Overall, 66.7% (66.6%–66.8%) of the children had two or more leading risk factors simultaneously (i.e., short birth interval, small birth size, never breastfed or delayed breastfeeding, low maternal education, and unsatisfied family planning needs). Additionally, we observed a high prevalence of concurrence in all 61 countries. For each individual country, there were more than 30% of children experiencing two or more leading risk factors. Moreover, our study explicitly demonstrates that the leading risk factors, when present simultaneous, have the most significant impact on child mortality in LMICs.

**Implications of all the available evidence**

The findings from our study provide further evidence that U5M remains a grave public health issue within LMICs. Aligned with the post-2015 Sustainable Development Agenda, we propose that accelerating progress in child health requires a coherent multi-sectoral approach of targeted health determinants to achieve reductions in child mortality. Our findings can be used by local, national, and global decision-makers to better understand the complexity of these simultaneous multiple leading risk factors that contribute to an elevated risk of U5M. This understanding can lead to more reliable, high-impact policy and intervention approaches to reduce the burden of U5M and improve child survival.

## Introduction

Reducing under-5 mortality (U5M) has been on top of the global health agenda for decades–the 2030 Sustainable Development Goals (SDGs) explicitly aim to achieve SDG 3.2, reducing under-five mortality rate (U5MR) to under 25 per 1000 live births worldwide by 2030.^1^ Despite substantial progress made over the past decades, the global burden of U5M remains high. In 2020, 5.2 million children worldwide died before reaching the age of five, with 2.4 millions of those deaths occurring in the newborn period.^2^ Income inequality across countries for children in their chances of survival continue to persist–the U5MR in low-income countries was 67 deaths per 1000 live births, which is 14 times higher than the 5 deaths per 1000 live births observed in high-income countries.^2^ At the country level, the U5MR (per 1000 live births) in 2021 ranged from 1.7 to 27.7 in high-income countries, while ranged from 2.3 to 115.2 for low- and middle-income countries (LMICs).^3^ LMICs bear the brunt of this mortality burden, accounting for over 80% of the total mortality, which has generally been attributed to the resource constraints and the frequent presence of risk factors.^2^ Accurately identifying high-risk populations in LMICs is therefore indispensable for allocating scarce resources and formulating effective policies to achieve the ambitious SDG targets and effectively reduce the preventable child deaths.

While previous studies^4^, ^5^, ^6^ have examined risk factors for child mortality in LMICs, for instance, sociodemographic characteristics, environmental factors, care-seeking behaviours, etc., they often overlook a crucial aspect: a substantial proportion of the children in LMICs are simultaneously exposed to multiple risk factors.^7^ Take Afghanistan as an example, it was estimated that nearly 20% of children are exposed to high indoor air pollution and maternal illiteracy simultaneously.^8^ Some previous literature attempted to assess the combined effects of health care practices and socioeconomic factors in some scattered settings, such as sub-Saharan Africa^9^ and Cambodia.^10^ However, these studies typically encompassed only two or three specific risk factors and were limited in capturing the complex reality faced by many children and households in LMICs.

In this study, we aim to systematically assess the concurrence of multiple risk factors across multiple countries in LMICs and, more importantly, to evaluate their association between the concurrent of multiple risk factors with U5M. By doing so, we aim to equip policymakers and program managers with actionable insights aligned with SDG 3.2, enabling them to effectively address the challenge of reducing U5M.^1^

## Methods

### Data source and data collection

This study is based on the most recent data for LMICs from Demographic and Health Surveys (DHS) platform conducted between January 1st, 2010, and December 31st, 2021. Surveys conducted before 2010 were excluded to maintain consistency in data collection and measurements across the surveys and the countries. We included 61 countries with available information regarding under-5 child survival status. The DHS are nationally representative surveys, which employ standardised questionnaires and follow a multistage stratified cluster sampling design to select the household from where data were collected.^8^ The target group for these surveys consisted of women aged 15–49 years who were residents of the selected households or had spent the previous night before the survey day at the selected households. For these selected women, their pregnancy history and the survival status of their children within the five years preceding the survey was recorded. Further detailed information about these surveys and the sample selection procedure can be found elsewhere.^8^ The World Bank Income Classification^11^ and Uppsala Conflict Data Program Georeferenced Event Dataset^12^ were also used to categorise all studied countries by income level and the presence of armed conflict events at the time of the survey (Appendix Table 1).

### Study sample

The eligibility criteria for our analytic sample were as follows: children (1) with valid information on the survival status, (2) being singleton, and (3) the last born birth to reduce reporting bias.^13^ To ensure consistency across countries and to minimise self-report bias, we only considered under-5 child deaths that occurred up to five years preceding the survey. Of the 851,255 children identified from the 61 LMICs, 246,883 were excluded. Specifically, 22,390 children were twins and therefore excluded, 224,490 were not the last-born child in their families, and an additional 3 children were excluded as their births did not fall within the specified five-year timeframe. Ultimately, a total of 604,372 children from 61 LMICs met these criteria and were included in this study. Based on the World Bank Income Classification,^11^ these 61 countries represent 45.5% of the 134 identified LMICs and encompass regions globally, except North America. The mean of U5MR (per 1000 live births) of other 73 non-DHS LMICs in 2015 was 35.1 (ranged from 2.8 to 126), whereas the included 61 LMICs showed a similar mean U5MR of 43.1 (ranged from 2 to 107; Appendix Table 1).^3^ See Appendix Table 1 for the number of observations for each country included in the study.

### Outcomes

The primary outcome of this study was U5M, defined as deaths from birth to 59 months. Additionally, four secondary outcomes were examined for children with recorded dates of death: neonatal mortality (deaths occurring from birth to 28 days), post-neonatal mortality (deaths occurring from 29 days to 364 days), infant mortality (deaths occurring from birth to 364 days), and childhood mortality (deaths occurring from 365 days to 59 months). For each outcome variable, we coded death case as 1, and 0 otherwise.

### Risk factors

We conducted a preliminary literature review to select potential risk factors by searching Google Scholar, Web of Science, and PubMed. Our search strategy used various combinations of terms: (“under-5 child mortality” [All Fields] OR “under-5 mortality” [All Fields] OR “under-5 child death” [All Fields] OR “child health” [All Fields]) AND (“risk factor” [All Fields] OR “causes” [All Fields] OR “intervention” [All Fields] OR “prevention” [All Fields] OR “identification” [All Fields]) AND “low-and middle-income countries [All Fields].” The selection was formed by the Mosley-Chen analytical framework,^14^ the framework of the Commission on Social Determinants of Health (CSDH),^15^ the global SDG indicator framework,^16^ and a systematic literature review regarding the causes of U5M.^6^ (see Appendix Table 2). For example, The Mosley-Chen framework conceptualises how socioeconomic determinants of child mortality in developing countries manifest through proximate determinants at the individual, household, and community level, including maternal factors (such as age and birth interval), environmental conditions (e.g., air and water quality, insect vectors), nutrient deficiencies, injury, and personal illness control measures.^14^ Moreover, CSDH highlights that health and mortality are influenced by both structural determinants and daily living conditions, encompassing aspects like education, healthcare, housing, and employment.^15^ Synthesizing these sources, we found the observable determinants of child mortality could be categorised into four domains, including household factors, parental factors, maternal care services, and child factors. Based on these and the data available in the DHS datasets, we included 20 observable factors for the four domains that have shown varying degrees of association with U5M in LMICs. There were five household factors, including household wealth, type of residence, drinking water source, sanitary facility, and indoor air pollution. The eight parental factors were current maternal marital status, maternal education, maternal occupation, maternal height, maternal age at childbirth, maternal smoking status, paternal occupation, and birth interval. We included five maternal care services indicators, which were skilled birth attendants at delivery, number of antenatal care visits, family planning need, tetanus toxoid injection during pregnancy. Three child-related indicators were included, which were breastfeeding initiation, birth order and birth size. A detailed list with definitions of all risk factors can be found in Table 1.

**Table 1:** Definition of 21 risk factors associated with under-5 mortality.

Risk factors	Definition	Reference category	Self-reported
**Household characteristics**			
Household wealth	Constructed by DHS based on a selected set of household assets through principal component analysis and classified in five equal quintiles: (1) poorest household wealth; (2) poorer; (3) middle; (4) richer; and (5) richest household wealth	Richest household wealth	Yes
Type of residence	Lives in the urban or rural, in the 2 following categories: (1) urban; (2) rural	Urban	No
Drinking water source	Safe if the household had access to water piped into dwelling, yard, or plot, public tap or standpipe, tube well or borehole, protected well or spring, rain water, and bottled water; unsafe otherwise	Safe water source	Yes
Sanitary facility	Improved if the household had access to flush to piped sewer system, septic tank or pit latrine, ventilated improved pit latrine, pit latrine with slab, and composting toilet; unimproved otherwise	Improved sanitation facility	Yes
Indoor air pollution	Low if the household used clean fuels for cooking (including electricity, liquid petroleum gas, natural gas and biogas); high otherwise (e.g., kerosene, coal, charcoal, wood, straw/grass, animal dung, agricultural crop)	Low indoor pollution	Yes
Household Possession of Mosquito Nets	Yes if the households with at least one mosquito net, no otherwise	Yes	Yes
**Parental characteristics**			
Current maternal marital status	In the 2 following categories: (1) married; (2) never or formerly married	Married	Yes
Maternal education	In the 5 following categories: (1) no schooling; (2) primary education; (3) secondary education; and (4) high school education; (5) higher education	Higher education	Yes
Maternal occupation	In the 3 following categories: (1) Not working/unknown; (2) working	Working	Yes
Maternal height	In the 2 following categories: (1) <145 cm; (2)≥145 cm; with <145 cm defined as short maternal height	≥145 cm	No
Maternal age at birth	Categorised as (1) 15–19 years old; (2) 20–34 years old; (3) 35–49 years old	20–34 years old	Yes
Maternal smoking status	Yes if the woman smoke, no otherwise	No smoking	Yes
Paternal occupation	In the 2 following categories: (1) Not working/unknown; (2) working	Working	Yes
Birth interval	The interval between the birth and the previously reported birth. In the 3 following categories: (1) < 18 months; (2) 18–59 month; (3) >59 months	18–59 months	Yes
**Maternal health care services received**			
Skilled birth attendants at delivery	Yes, if a woman delivered the child with skilled birth attendant (within or outside the formal healthcare facilities), including physicians, nurses, and midwives; no otherwise	With skilled birth attendant	Yes
Number of antenatal care visits	Number of antenatal care visits from a skilled provider for the most recent birth. Categorised as: (1) <4 antenatal care visits; (2) ≥4 antenatal care visits	≥4 antenatal care visits	Yes
Family planning need	Satisfied if the woman, who was fecund and married or in a union, wishes to stop or delay childbearing or is currently using any modern method of contraception; unsatisfied otherwise	Family planning need satisfied	Yes
Tetanus toxoid injection during pregnancy	Women received tetanus toxoid injection during pregnancy. In the following 3 categories: (1) received no injection; (2) 1 injection; (3) 2 or more than 2 injections	2 or more than 2 injections	Yes
**Child characteristics**			
Breastfeeding initiation	In the 2 following categories: (1) initiation of breastfeeding within 1 h of birth; (2) never breastfed or initiation of breastfeeding > 1 h of birth, defined as delayed breastfeeding	Breastfeeding initiation <1 h of birth	Yes
Birth order	Birth order of the child, in the 4 following categories: (1) 1st; (2) 2nd or 3rd; (3) 4th or 5th; (4) 6th and above	First child	Yes
Birth size	The baby's size at time of birth perceived by mother. In the 5 following categories: (1) very large; (2) larger than average; (3) average; (4) smaller than average; (5) very small	Average	Yes

Note: For all risk factors, the better-off group set as the reference category in our analysis to ensure consistency when interpreting the odds ratios (ORs). We only present the ORs of the worst-off group in our results.

### Statistics

We assessed the association between the risk factors and U5M. All analysis considered sampling weights, strata, and primary sampling units provided by DHS. In line with previous practices, we included country-fixed effects by developing a binary variable for each country to account for the unobservable country-level factors in our analyses.^17^

Before performing multivariable analysis, the multicollinearity between variables was assessed using variance inflation factor (VIF), and all variables exhibited low VIF values (<3), indicating lack of presence of multicollinearity (Appendix Table 3). Subsequently, we constructed two sets of binary logistic regression models: first, we conducted single adjusted logistic regression models to estimate the association between each risk factor and U5M, adjusting for child sex, child age, and country-fixed effects. Secondly, we executed a mutually-adjusted regression model that included all risk factors simultaneously, with adjustments for child sex, age, and country-fixed effects. For all risk factors, the better-off group set as the reference category in our analysis to ensure consistency when interpreting the odds ratios (OR). For variables with multiple categories, the ORs of the worst-off group were presented in our results.^17^ We identified the factors with ORs greater than 1 as the risk factors of U5M. Following previous practice,^18^ coefficient sizes from the mutually-adjusted regression models were recorded and ordered, and risk factors with significant coefficient sizes were selected as the leading factors associated with U5M.

We adopted the well-established analytical method developed by previous studies^18^, ^19^, ^20^, ^21^ and examined the cumulative effects of multiple leading risk factors coexisting on U5M with two different approaches: first, the concurrence of multiple risk factors was calculated by counting the number of presence of the five primary risk factors for each individual. For example, an individual possessing any two of the five identified leading risk factors simultaneously would be identified as the concurrence of two risk factors. Logistic regression was then applied to investigate the effects of concurrence of risk factors on U5M, adjusting for child sex, age, and country-fixed effects. Second, we followed the previous practice and constructed a weighted risk score based on the magnitude of the regression coefficients (βs) from the leading risk factors.^18^^,^^19^ Each individual was assigned a weighted risk score by summing up the scores for the top 5 risk factors. We then examined the association between the weighted risk scores and U5M with mutually-adjusted logistic regression model, adjusting for child sex, age, and country-fixed effects. Observations with a risk score of 0 served as the reference group for this analysis. We carried out multiple imputation for observations with missing values on one or more risk factors of interest in all pooled analyses, using chained equations and logistic regression model.

We performed five additional subgroup analyses. First, considering that the ownership of mosquito nets within households is primarily relevant to prevalent malaria,^22^^,^^23^ we have included household mosquito net possession as a risk factor to examine the relative importance of risk factors in our stratified analysis of malaria-endemic countries.^24^ Second, to investigate whether the association differed by child sex, we examined the relative importance of risk factors according to child sex using mutually-adjusted logistic regression. Third, missing values were treated as separate categories to further test the consistency of the findings, again employing mutually-adjusted logistic regression. Fourth, based on the World Bank income classification,^11^ we categorised all included countries into 3 groups by countries’ income levels (i.e., low income, lower-middle income, and upper-middle income) to examine the associations between the concurrence of multiple leading risk factors and U5M. Fifth, we investigated whether the presence of armed conflict events affects the cumulative effects of multiple leading risk factors on U5M, considering the extensive literature demonstrating their detrimental influence on child survival, including malnutrition, physical injuries, infectious diseases, and even direct deaths.^25^^,^^26^ To examine this association, we utilised the Uppsala Conflict Data Program Georeferenced Event Dataset,^12^ a comprehensive resource documenting violence and conflict events globally since 1946. We analysed this relationship by stratifying countries based on their armed conflict status at the time of the survey.

To test the robustness of our results, we performed five sets of sensitivity analysis: First, to explore if the simultaneous presence of an increasing number of other risk factors was linked to U5M, we extended our investigation beyond the top five risk factors. This was accomplished by incorporating all risk factors that exhibited significant associations with U5M, as determined from mutually-adjusted logistic regression models. Second, we assessed the association between the concurrence of multiple leading risk factors and U5M by excluding a total of 28 countries where observations on one or more of the included risk factors were not available (Appendix Table 4). As a result, this sensitivity analysis included a subset of 409,769 children from 33 countries. Third, in order to ensure our results were not affected by multiple imputation applied to the missingness, we conducted a subsequent sensitivity analysis excluding the observations with missing values on one or more risk factors. Fourth, there was a large difference (across a 10-year period) in the timing of our surveys. To control for the effects of time, we conducted a sensitivity analysis that included the year-fixed effects into our model to examine the association between the concurrence of leading risk factors and U5M. Fifth, we included all children without imposing any exclusion criteria to examine the association between the concurrence of leading risk factors and U5M.

We followed the Strengthening the Reporting of Observational Studies in Epidemiology (STROBE) reporting guideline. All statistical analyses were conducted by using STATA (version 17.0) and descriptive analyses were assessed with two-sided tests. A *p* < 0.05 was used to establish statistical significance.

### Ethics

The DHS maintained strict ethical standards of anonymity, confidentiality, and informed consent. Our research protocol was submitted to the DHS, where we gained authorization to access and analyze the datasets. This study was reviewed and approved by Tsinghua Ethics Institutional Review Board (Project No. 20220005), and given the nature of the use of secondary data in our study, informed consent was waived by Tsinghua Ethics Institutional Review Board. This study followed the guidelines of Strengthening the Reporting of Observational Studies for cross-sectional studies.

### Role of funding source

The funders of the study had no role in study design, data collection, data analysis, data interpretation, or writing of the report.

## Results

A total of 604,372 under-5 children from 61 LMICs were included in the study, with a mean age of 24.3 (standard deviation ± 16.4) months, and 290,154 (48.0%) were females. Of these, 586,206 were alive and 18,166 (3.0%) had died at the time of the survey. In total, 148,708 (24.6%) children were from the poorest households, and 414,841 (68.6%) resided in rural areas. Additionally, dead children were more likely to come from poor households, rural areas, lack access to safe drinking water, use unimproved sanitary facilities, and be exposed to indoor air pollution. Furthermore, dead children were more likely to born to the mothers with low education, fewer number of antenatal care visits, unmet family planning needs, and lack skilled birth attendants at delivery (Table 2).

**Table 2:** Summary table of the sample characteristics.

	Total, No. (%)	Alive children, No. (%)	Dead children, No. (%)	*p*-value
**Total number of observations**	604,372	586,206 (97.0%)	18,166 (3.0%)	
***Household characteristics***				
**Household Wealth**				<0.001
Poorest	148,708 (24.6%)	143,538 (24.5%)	5170 (28.5%)	
Poorer	133,346 (22.1%)	128,890 (22.0%)	4456 (24.5%)	
Middle	119,474 (19.8%)	115,945 (19.8%)	3529 (19.4%)	
Richer	108,912 (18.0%)	105,940 (18.1%)	2972 (16.4%)	
Richest	93,932 (15.5%)	91,893 (15.6%)	2039 (11.2%)	
**Place of residence**				<0.001
Urban	189,531 (31.4%)	184,738 (31.5%)	4793 (26.4%)	
Rural	414,841 (68.6%)	401,468 (68.5%)	13,373 (73.6%)	
**Safe drinking water source**				<0.001
No	131,927 (21.8%)	126,906 (21.7%)	5021 (27.6%)	
Yes	456,957 (75.6%)	444,189 (75.8%)	12,768 (70.3%)	
Missing	15,488 (2.6%)	15,111 (2.5%)	377 (2.1%)	
**Improved sanitary facility**				<0.001
No	231,883 (38.4%)	223,046 (38.0%)	8837 (48.6%)	
Yes	344,263 (57.0%)	335,867 (57.3%)	8396 (46.2%)	
Missing	28,226 (4.6%)	27,293 (4.7%)	933 (5.2%)	
**Indoor air pollution**				<0.001
No	418,788 (69.3%)	404,024 (68.9%)	14,764 (81.2%)	
Yes	171,363 (28.4%)	168,136 (28.7%)	3227 (17.8%)	
Missing	14,221 (2.4%)	14,046 (2.4%)	175 (1.0%)	
**Household Possession of Mosquito Nets**				<0.001
No	254,984 (42.2%)	250,581 (42.7%)	4403 (24.2%)	
Yes	193,226 (32.0%)	190,347 (32.5%)	2879 (15.8%)	
Missing	156,162 (25.8%)	145,278 (24.8%)	10,884 (60.0%)	
***Parental characteristics***				
**Current maternal marital status**				<0.001
Formerly or never married	95,640 (15.8%)	92,421 (15.8%)	3219 (17.7%)	
Married	484,606 (80.2%)	470,574 (80.2%)	14,032 (77.3%)	
Missing	24,126 (4.0%)	23,211 (4.0%)	915 (5.0%)	
**Maternal education**				<0.001
No schooling	172,130 (28.5%)	164,944 (28.1%)	7186 (39.6%)	
Primary	101,498 (16.8%)	98,055 (16.7%)	3443 (19.0%)	
Secondary	194,249 (32.0%)	189,048 (32.2%)	5201 (28.5%)	
High school	78,285 (13.0%)	76,783 (13.2%)	1502 (8.3%)	
Higher	57,899 (9.6%)	57,069 (9.7%)	830 (4.6%)	
Missing	311 (0.1%)	307 (0.1%)	4 (0.0%)	
**Maternal occupation**				<0.001
Unemployed	207,102 (34.3%)	201,274 (34.3%)	5828 (32.1%)	
Employed	240,683 (39.8%)	232,083 (39.6%)	8600 (47.3%)	
Missing	156,587 (25.9%)	152,849 (26.1%)	3738 (20.6%)	
**Maternal height**				<0.001
≥145 cm	366,334 (60.6%)	356,269 (60.8%)	10,065 (55.4%)	
<145 cm	28,982 (4.8%)	28,007 (4.8%)	975 (5.4%)	
Missing	209,056 (34.6%)	201,930 (34.4%)	7126 (39.2%)	
**Maternal age at birth**				<0.001
≤19	51,566 (8.5%)	49,610 (8.5%)	1956 (10.8%)	
20–34	459,126 (76.0%)	447,011 (76.2%)	12,115 (66.7%)	
≥35	93,680 (15.5%)	89,585 (15.3%)	4095 (22.5%)	
**Maternal smoking status**				<0.001
No	531,616 (87.9%)	515,437 (88.0%)	16,179 (89.1%)	
Yes	6365 (1.1%)	6103 (1.0%)	262 (1.4%)	
Missing	66,391 (11.0%)	64,666 (11.0%)	1725 (9.5%)	
**Paternal occupation**				0.64
Unemployed	22,537 (3.7%)	21,855 (3.7%)	682 (3.8%)	
Employed	371,454 (61.5%)	359,463 (61.3%)	11,991 (66.0%)	
Missing	210,381 (34.8%)	204,888 (35.0%)	5493 (30.2%)	
**Birth interval**				<0.001
<18 months	31,588 (5.2%)	29,844 (5.1%)	1744 (9.6%)	
18–59 months	323,642 (53.6%)	313,940 (53.5%)	9702 (53.4%)	
>59 months	249,142 (41.2%)	242,422 (41.4%)	6720 (37.0%)	
***Maternal care received during pregnancy***				
**Skilled birth attendant at delivery**				<0.001
No	164,456 (27.2%)	157,815 (26.9%)	6641 (36.5%)	
Yes	437,031 (72.3%)	425,680 (72.6%)	11,351 (62.5%)	
Missing	2885 (0.5%)	2711 (0.5%)	174 (1.0%)	
**Number of antenatal care visit**				<0.001
Less than 4 times	239,237 (39.6%)	230,003 (39.2%)	9234 (50.8%)	
At least 4 times	353,789 (58.5%)	345,147 (58.9%)	8642 (47.6%)	
Missing	11,346 (1.9%)	11,056 (1.9%)	290 (1.6%)	
**Family planning need satisfied**				<0.001
No	329,149 (54.5%)	315,816 (53.9%)	13,333 (73.4%)	
Yes	266,458 (44.0%)	261,888 (44.7%)	4570 (25.2%)	
Missing	8765 (1.5%)	8502 (1.4%)	263 (1.4%)	
**Number of tetanus toxoid injection during pregnancy**				<0.001
Never	118,371 (19.5%)	113,575 (19.4%)	4796 (26.4%)	
1	114,130 (18.9%)	110,773 (18.9%)	3357 (18.5%)	
≥2	342,528 (56.7%)	333,158 (56.8%)	9370 (51.6%)	
Missing	29,343 (4.9%)	28,700 (4.9%)	643 (3.5%)	
***Child characteristics***				
**Breastfeeding initiation**				<0.001
Within 1 h of birth	301,307 (49.9%)	295,139 (50.3%)	6168 (34.0%)	
Never breastfed or delayed breastfeeding	281,425 (46.6%)	270,088 (46.1%)	11,337 (62.4%)	
Missing	21,640 (3.5%)	20,979 (3.6%)	661 (3.6%)	
**Birth order**				<0.001
1st	161,396 (26.7%)	156,885 (26.8%)	4511 (24.8%)	
2nd to 3rd	255,202 (42.2%)	249,228 (42.5%)	5974 (32.9%)	
4th to 5th	108,030 (17.9%)	104,399 (17.8%)	3631 (20.0%)	
6th or above	79,744 (13.2%)	75,694 (12.9%)	4050 (22.3%)	
**Birth size**				<0.001
Very large	45,672 (7.6%)	44,022 (7.5%)	1650 (9.1%)	
Larger than average	102,823 (17.0%)	99,812 (17.0%)	3011 (16.6%)	
Average	326,658 (54.0%)	318,495 (54.3%)	8163 (44.9%)	
Smaller than average	60,428 (10.0%)	58,192 (10.0%)	2236 (12.3%)	
Very small	24,342 (4.0%)	22,751 (3.9%)	1591 (8.8%)	
Missing	44,449 (7.4%)	42,934 (7.3%)	1515 (8.3%)	

### Identification of the leading risk factors with pooled analyses

**Fig. 1:**
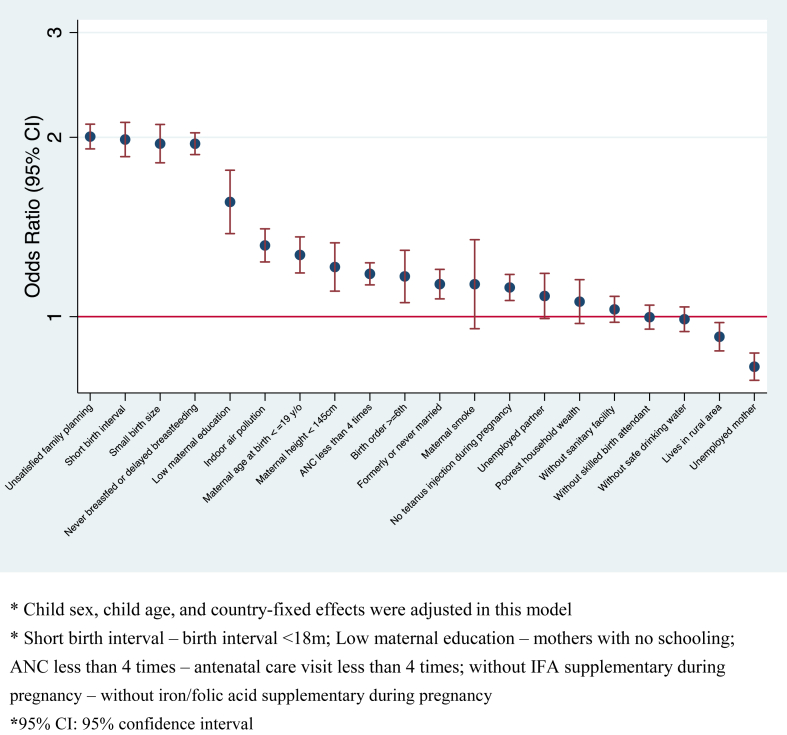
Relative ranking of risk factors associated with under-5 mortality from mutually-adjusted models, pooled analysis of 61 countries (N = 604,372).

We presented the results of pooled analyses using mutually-adjusted regression model in Fig. 1. Our model showed high predictive power with an area under the ROC Curve of 0.83 (Appendix Fig. 1). Unsatisfied family planning needs had the strongest association with U5M, with an OR of 2.0 and 95% confidence interval (CI) of 1.9–2.1, followed by short birth interval (<18 months; OR: 2.0, 95% CI: 1.9–2.1), small birth size (OR: 2.0, 95% CI: 1.8–2.1), never breastfed or delayed breastfeeding (OR: 2.0, 95% CI: 1.9–2.0), and low maternal education (OR: 1.6, 95% CI: 1.4–1.8). The results from single logistic regression were generally consistent with the mutually-adjusted results with larger coefficient magnitudes. For example, the coefficient of low maternal education in the single-adjusted model was 2.8 (95% CI: 2.5–3.2), which attenuated to 1.6 (95% CI: 1.4–1.8) in the mutually-adjusted model (Appendix Fig. 2). Our analyses concerning the secondary outcomes were also partially consistent with the overall analyses (Appendix Fig. 3).

### The prevalence of concurrence of multiple leading risk factors and their associations with U5M

Upon identification of the top-five risk factors showing the strongest associations with U5M (i.e., short birth interval, low birth weight, low maternal education, formerly or never married, and unsatisfied family planning needs), we found that out of the total 604,372 children, there were 66.7% (66.6%–66.8%) children experiencing 2 or more of these risk factors simultaneously (Table 3). Country-specific prevalence of concurrence of multiple leading risk factors were presented in Appendix Table 5. The prevalence of concurrence of 2 or more leading risk factors varied from 36.6% (95% CI: 35.4%–37.8%) in Rwanda to 94.8% (95% CI: 94.3%–95.2%) in Chad. The other countries with the highest prevalence of co-existing leading risk factors were Maldives (94.5%, 95% CI: 93.6%–95.4%), Madagascar (94.0%, 95% CI: 63.0%–65.0%), Afghanistan (90.3%, 95% CI: 89.9%–90.7%), and Burkina Faso (90.0%, 95% CI: 89.3%–90.6%). Among the 61 LMICs examined, all 61 exhibited a prevalence exceeding 30%. Moreover, 51 of these LMICs, demonstrated a prevalence of concurrent risk factors surpassing 50%. Regionally, the highest prevalence of concurrence of 2 or more leading risk factors was found in sub-Saharan African (70.8%, 95% CI: 70.6%–71.0%), followed by South Asia (65.7%, 95% CI: 65.5%–65.9%). In terms of income level, low-income countries showed the highest prevalence, with 71.0% (95% CI: 70.8%–71.2%) of the children having 2 or more leading risk factors simultaneously (Appendix Table 6).

**Table 3:** Prevalence (95% Confidence Interval; CI) of children with concurrence of 2 or more risks factors.

	Alive children, prevalence (95% CI)	Dead children, prevalence (95% CI)	All children, prevalence (95% CI)
**Overall**	66.2% (66.1%–66.3%)	83.1% (82.6%–83.7%)	66.7% (66.6%–66.8%)
**By income groups**			
Low-income countries	70.5% (70.2%–70.7%)	83.8% (82.9%–84.7%)	71.0% (70.8%–71.2%)
Lower-middle income countries	65.5% (65.4%–65.7%)	83.5% (82.8%–84.2%)	66.0% (65.9%–66.2%)
Upper-middle income countries	59.2% (58.8%–59.6%)	74.2% (71.3%–76.8%)	59.5% (59.1%–59.9%)
**By region groups**			
East Asia and Pacific	59.7% (59.3%–60.2%)	81.4% (78.8%–83.7%)	60.2% (59.8%–60.7%)
Europe and Central Asia	54.6% (53.7%–55.4%)	80.5% (73.4%–86.1%)	54.8% (54.0%–55.7%)
Latin America and the Caribbean	60.7% (60.2%–61.2%)	74.3% (71.2%–77.1%)	61.0% (60.5%–61.4%)
Middle East and North Africa	61.9% (61.3%–62.5%)	85.5% (82.4%–88.2%)	62.4% (61.8%–62.9%)
South Asia	65.2% (65.0%–65.4%)	84.3% (83.3%–85.2%)	65.7% (65.5%–65.9%)
Sub-Saharan Africa	70.3% (70.1%–70.5%)	83.3% (82.6%–84.1%)	70.8% (70.6%–71.0%)

Note: The concurrence of multiple risk factors was computed by counting the presence of the top 5 risk factors (i.e., unsatisfied family planning, short birth interval (<18 months), small birth size, never breastfed or delayed breastfeeding, and low maternal education (no schooling)) for each individual.

Additionally, we presented the percentages of children died by the number of the concurrence of leading risk factors in Appendix Table 7. There was a progressive increase in the proportion of U5M with the escalating number of co-existing leading risk factors–the percentage of deaths increased from 1.1% (95% CI: 1.0%–1.2%) among children with no risk factor to 9.3% (95% CI: 8.4%–10.1%) for those experiencing a concurrence of all 5 risk factors. Similar increasing trends were also found in different regions and income levels.

**Fig. 2:**
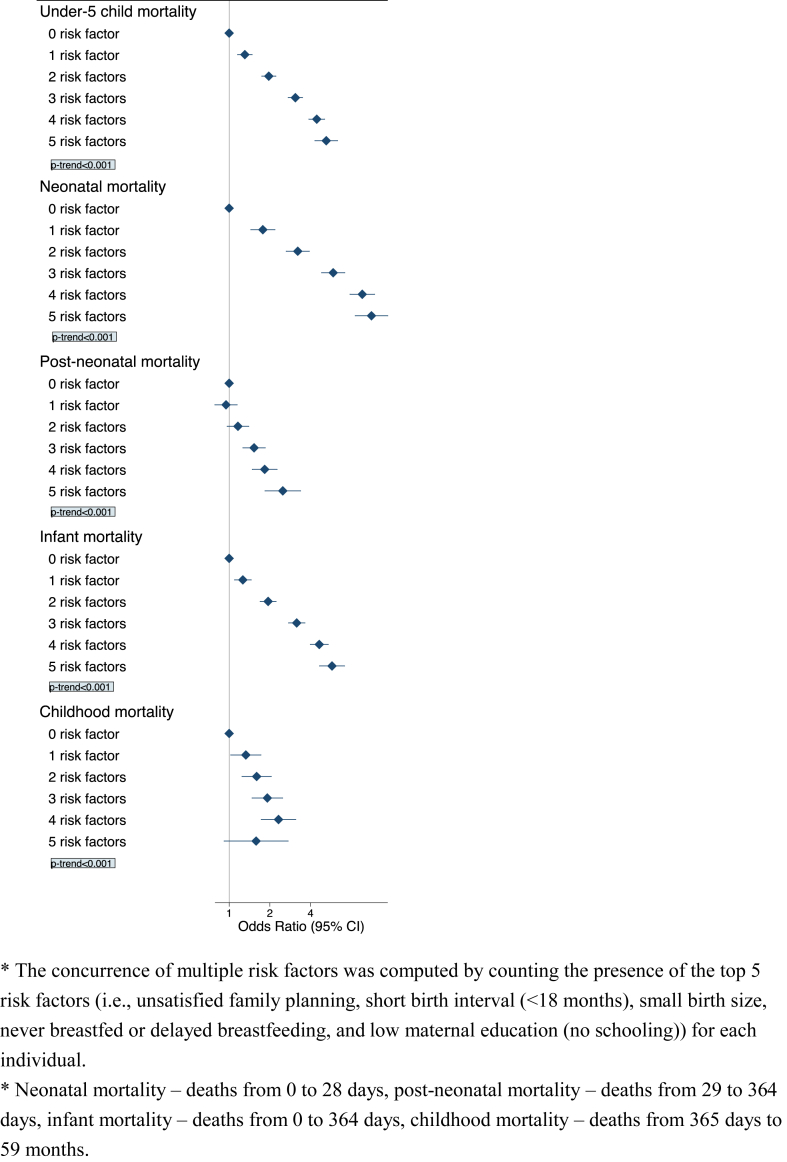
Associations between concurrence of multiple leading risk factors and under-5 mortality, neonatal mortality, post-neonatal mortality, infant mortality, and childhood mortality; odds ratio (ORs) and 95% confidence interval (CI).

We showed the associations between concurrence of multiple leading risk factors and U5M in Fig. 2. Compared with children having no leading risk factor, the odds of U5M with 1 risk factor was 1.3 (95% CI: 1.2–1.5). Our analysis portrayed there was an increased OR corresponding to the presence of each additional risk factor. For example, children who suffered from 2 leading risk factors simultaneously had 2.0 (95% CI: 1.7–2.2) times higher odds of U5M compared to those with no leading risk factor. For children who showed concurrence of all 5 leading risk factors, the OR of U5M was 5.2 times higher (95% CI: 4.3–6.3). A dose–response relationship was also observed between the concurrence of risk factors and U5M− as each incremental presence of leading risk factors increased, a corresponding rise in U5M was noted (*p-trend* < 0.001). Analyses on secondary outcomes (i.e., neonatal mortality, post-neonatal mortality, infant mortality, and childhood mortality) presented similar results. For example, children during the neonatal period who were subjected to have all 5 leading risk factors exhibited a significantly elevated odds of U5M (OR: 11.3, 95% CI: 8.6–14.9). In terms of our weighted risk score findings, children with higher risk scores demonstrated a similar rising trend of U5M (see Appendix Table 8). Children scored 6 or higher showed an OR of U5M of 4.0 (95% CI: 3.5–4.5). Comparable increasing trends were also observed for all secondary outcomes including neonatal mortality, post-neonatal mortality, infant mortality, and childhood mortality (*p-trend* <0.001 for all; Appendix Fig. 4).

### Stratified analysis

For our first stratified analysis, we included the household possession of mosquito bed net as a risk factor for countries with malaria endemic. We found that without mosquito bed net in households was significantly associated with U5M, with an OR of 1.1 (95% CI: 1.0–1.1). Nevertheless, it was not identified as leading risk factors with a lower rank of 15 (Appendix Fig. 5). The results of stratified analyses by sex of the children and countries’ income level were largely consistent with the main results (Appendix Figs. 6 and 7). The results of treating missing values as separate categories showed consistency with our primary findings (Appendix Fig. 8). In the last stratified analysis (Appendix Fig. 9), In both countries without armed conflict and those suffering such conflicts, children with all 5 major risk factors exhibited a substantial connection with U5M with an OR of 6.1 (95% CI: 4.8–7.9) and OR of 4.1 (95% CI: 3.1–5.4), respectively.

Significant associations between concurrence of multiple leading risk factors and U5M was identified in the sensitivity analysis including all significant 16 risk factors (Appendix Fig. 10). In analysis excluding the 28 countries lacking observation data for one or more risk factors of interest, we found similar increasing trends for the remaining 33 countries (Appendix Fig. 11). The results of the dataset without multiple imputation were generally consistent with the main analysis (Appendix Fig. 12). The upward trends persisted in the sensitivity analysis that adjusted the year-fixed effects (Appendix Fig. 13). As the results of including all children without applying any exclusion criteria, the similar increasing trend was also observed (Appendix Fig. 14).

## Discussion

Our analysis of 604,372 children from 61 LMICs spanning the period from 2010 to 2021 yielded three salient findings. Firstly, 5 leading risk factors associated U5M were identified in our study, including short birth interval (<18 months), small birth size, never breastfed or delayed breastfeeding, low maternal education, and unsatisfied family planning needs. Secondly, there was a high prevalence of the co-occurrence of two or more risk factors among children in LMICs. Notably, in 51 countries, more than 50% of the children experienced the simultaneous presence of at least two of the top-five prominent risk factors associated with U5M. Thirdly, individuals with an increasing number of concurrent leading risk factors had significantly higher risk of U5M.

The robust correlation observed in this study between reproductive behaviours (such as birth intervals and family planning), small birth size, breastfeeding initiation, and maternal education, with U5M, has garnered substantial validation from preceding studies.^27^^,^^28^ For instance, a meta-analysis conducted by the World Health Organization that focused on the determinants of child health, leveraging studies using DHS datasets have addressed the pivotal role of birth intervals and maternal education in shaping the survival prospects of child health.^29^ As the detrimental effects of short birth intervals (<18 months), we speculated the possibly may contribute to severe maternal health complications due to insufficient recovery time from post-partum, compromising children's nutritional and physical development in utero and early life.^27^^,^^30^ As such, our study highlights the crucial need for policy interventions aimed at improving gender equity and free maternal care, especially in resource-poor settings. This approach not only supports maternal health and empowerment but also contributes to the sustainable development goals by reducing child mortality and improving maternal health.

In light of the substantial number of children in LMICs facing multiple risk factors, our research has implied that U5M in LMICs might frequently results from the convergency of multiple risk factors rather than a single cause. Despite the absence of prior research on LMICs delving into this specific topic, our findings align with established studies on the cumulative impact of risk factors on child health. Research endeavors focused on stillbirths, for instance, have illuminated the profound effects of multiple risk factors (e.g., short maternal height, low socioeconomic status, and previous stillbirth history), in shaping stillbirths outcome.^18^ Another study conducted in United States also stressed the significant association between cumulative social disadvantages, including poverty, low parental education, and minority ethnicity, with adverse child health.^31^ These collective insights emphasise the urgency for a comprehensive and integrated approach towards child health policy and intervention design, especially for populations vulnerable to the concurrence of struggle with multiple risk factors at once.

Interventions that target multiple risk factors simultaneously hold potential for improving child well-being in LMICs. In line with the SDGs 17 of “partnerships for the goals,” multisectoral strategies and service delivery programs were also increasingly implemented and promoted beyond the remit of health sector. Notable examples include Rwanda's Vision 2020 program developed an intersectoral strategy (e.g., quality of primary care service and family planning coverage) to address multiple health determinants simultaneously.^32^ This integrated program led to a remarkable 67% decline in U5M from 2000 to 2015.^32^ Chile has also made noteworthy progress on child health through the comprehensive child protection program Chile Crece Contigo, emphasizing the delivery of multisectoral interventions covering various domains related to children's growth and development.^33^ The sustainability of this program lies in the interdependence of the health, social protection, and education sectors, delivered through networks of intersectoral teams from national to local levels.^33^ These examples underscore the imperative role of multisectoral policy approaches in effecting substantial improvements in child health and well-being. By acknowledging the interplay of diverse risk factors and adopting a holistic approach through collaborative efforts, we can pave the way for a more promising and healthier future for the most vulnerable populations in LMICs.

The study has several limitations. First, the findings may not be generalizable to the global level or specific income groups since the study only included data from 61 LMICs with available data. Even though DHS data contains data from all major global regions, cautions should be exercised when applying the results to other settings. Second, given the cross-sectional nature of our data, results presented here should not be given any causal interpretation. Third, some indicators in our study (e.g., reproductive behaviour) were collected through self-reported questions based on respondents' recall, and might lead to recall bias.^34^ Fourth, our study utilised the wealth quintile provided by the DHS as a measure of household wealth. We acknowledged that this approach may not fully capture the relative wealth status across different countries, especially when comparing individuals from lower-income countries with those from higher-income countries.^35^ Despite this limitation, the use of wealth quintiles is crucial for evaluating within-country equity and relative economic poverty, as it is tailored to each country's specific economic context at the time of the survey. Nevertheless, this limitation is important to consider when interpreting the results of our study. Last, we were unable to include some other related variables in our study. For example, vaccination as a risk factor were excluded in our study due to the missing information in dead children, which is a common risk factor of child mortality.^36^ Besides, exclusive breastfeeding was also not included as a factor due to the absence of retrospective breastfeeding data for children aged six months to five years in the DHS dataset, underscoring a gap in assessing its impact on under-5 mortality. Moreover, we did not account for the risk factor of use of iron and folic acid supplements during pregnancy.^37^ This omission is attributed to the lack of available data in over 10 countries (e.g., Zimbabwe, Turkey, and South Africa) and substantial missing values exceeding 30% in many countries (e.g., India, Bangladesh) within our dataset. This study provides new insights and contributes to the knowledge base by examining the correlation between concurrence of risk factors and U5M. Our results on the strong association between U5M and reproductive behaviour, household conditions, and maternal education highlight the importance of developing integrated, importance multisectoral policy and intervention strategies for further reduction in U5M.

## Contributors

YK, ZL, SC, and NM had full access to all of the data in the study and take responsibility for the integrity of the data and the accuracy of the data analysis. ZL and YK conceptualized and designed the study. YK, SC, NM, NK, GS, GF, PF, and ZL wrote the initial manuscript. YK, SC, NM, ZC, PK, GF, FW, DW, MZ, SMG, NK, AR, LR, JA, PF, ZL contributed to the data analysis and interpretation of the results. YK, SC, XK, LF, GS, PG, FC, JN, PF and ZL contributed to the writing. All authors contributed to the critical revision of the manuscript for important intellectual content. ZL provided overall supervision of the study. All of the authors approved the final submission of the study.

## Data sharing statement

The data used in this study is publicly available and can be obtained after requesting from DHS (https://dhsprogram.com/).

## Declaration of interests

The authors declare no competing interests.

## References

[1] Sachs J., Kroll C., Lafortune G., Fuller G., Woelm F. (2021).

[2] UNICEF Levels and trends in child mortality 2020. https://data.unicef.org/resources/levels-and-trends-in-child-mortality/.

[3] Bank W. (2022). https://data.worldbank.org/indicator/SH.DYN.MORT?locations=XO&most_recent_year_desc=false.

[4] Günther I., Fink G. (2011).

[5] Anyamele O.D., Ukawuilulu J.O., Akanegbu B.N. (2017). The role of wealth and Mother's education in infant and child mortality in 26 sub-Saharan African countries: evidence from pooled demographic and health survey (DHS) data 2003–2011 and African development indicators (ADI), 2012. Soc Indicat Res.

[6] Liu L., Johnson H.L., Cousens S. (2012). Global, regional, and national causes of child mortality: an updated systematic analysis for 2010 with time trends since 2000. Lancet.

[7] Grantham-McGregor S., Cheung Y.B., Cueto S. (2007). Developmental potential in the first 5 years for children in developing countries. Lancet.

[8] USAID The DHS program–available datasets. https://www.dhsprogram.com/data/available-datasets.cfm.

[9] Akinyemi J.O., Adedini S.A., Wandera S.O., Odimegwu C.O. (2016). Independent and combined effects of maternal smoking and solid fuel on infant and child mortality in sub-Saharan Africa. Trop Med Int Health.

[10] Meessen B., Bigdeli M., Chheng K. (2011). Composition of pluralistic health systems: how much can we learn from household surveys? An exploration in Cambodia. Health Pol Plann.

[11] Bank W. (2020).

[12] Sundberg R., Melander E. (2013). Introducing the UCDP georeferenced event dataset. J Peace Res.

[13] Rutstein S.O. (2014).

[14] Mosley W.H., Chen L.C. (1984). An analytical framework for the study of child survival in developing countries. Popul Dev Rev.

[15] Solar O., Irwin A. (2010).

[16] UNICEF (2018).

[17] Li Z., Kim R., Vollmer S., Subramanian S. (2020). Factors associated with child stunting, wasting, and underweight in 35 low-and middle-income countries. JAMA Netw Open.

[18] Li Z., Kong Y., Chen S. (2022). Independent and cumulative effects of risk factors associated with stillbirths in 50 low-and middle-income countries: a multi-country cross-sectional study. eClinicalMedicine.

[19] Rawshani A., Rawshani A., Franzén S. (2018). Risk factors, mortality, and cardiovascular outcomes in patients with type 2 diabetes. N Engl J Med.

[20] Willcox B.J., He Q., Chen R. (2006). Midlife risk factors and healthy survival in men. JAMA.

[21] Pantell M.S., Prather A.A., Downing J.M., Gordon N.P., Adler N.E. (2019). Association of social and behavioral risk factors with earlier onset of adult hypertension and diabetes. JAMA Netw Open.

[22] Konlan K.D., Kossi Vivor N., Gegefe I., Hayford L. (2022). Factors associated with ownership and utilization of insecticide treated nets among children under five years in sub-Saharan Africa. BMC Publ Health.

[23] Malaria R.B. (2005).

[24] Bank W. (2022). https://data.worldbank.org/indicator/SH.MLR.INCD.P3?locations=XN.

[25] Bendavid E., Boerma T., Akseer N. (2021). The effects of armed conflict on the health of women and children. Lancet.

[26] Wagner Z., Heft-Neal S., Bhutta Z.A., Black R.E., Burke M., Bendavid E. (2018). Armed conflict and child mortality in Africa: a geospatial analysis. Lancet.

[27] Canning D., Schultz T.P. (2012). The economic consequences of reproductive health and family planning. Lancet.

[28] Wardlaw T.M. (2004).

[29] Charmarbagwala R., Ranger M., Waddington H., White H. (2004).

[30] Conde-Agudelo A., Rosas-Bermudez A., Kafury-Goeta A.C. (2007). Effects of birth spacing on maternal health: a systematic review. Am J Obstet Gynecol.

[31] Bauman L.J., Silver E.J., Stein R.E. (2006). Cumulative social disadvantage and child health. Pediatrics.

[32] Kaberuka D. (2000).

[33] Milman H.M., Castillo C.A., Sansotta A.T., Delpiano P.V., Murray J. (2018). Scaling up an early childhood development programme through a national multisectoral approach to social protection: lessons from Chile Crece Contigo. BMJ.

[34] Ties Boerma J., Sommerfelt A.E. (1993). Demographic and health surveys (DHS: contributions and limitations. World Health Stat Q.

[35] Mayfour K.W., Hruschka D. (2022). Assessing comparative asset-based measures of material wealth as predictors of physical growth and mortality. SSM Popul Health.

[36] Aaby P., Rodrigues A., Kofoed P.-E., Benn C.S. (2015). RTS,S/AS01 malaria vaccine and child mortality. Lancet.

[37] Nisar Y.B.D.M. (2016). Iron/folic acid supplementation during pregnancy prevents neonatal and under-five mortality in Pakistan: propensity score matched sample from two Pakistan Demographic and Health Surveys. Glob Health Action.

