# Supplementary material for: Association between concurrence of multiple risk factors and under-5 mortality: a pooled analysis of data from Demographic and Health Survey in 61 low-and-middle-income countries
Source: eClinicalMedicine. 2024 Apr 5;71:102583. doi: 10.1016/j.eclinm.2024.102583 (PMC11015335; doi:10.1016/j.eclinm.2024.102583)
Supplement: Appendix Figures and Tables [file mmc1.docx]

**APPENDIX**

**Appendix Table 1.** Countries included in the study

**Appendix Table 2.** Selection of risk factors

**Appendix Table 3.** Variance inflation factor (VIF) for the risk factors

**Appendix Table 4.** Countries with no observation on one or more risk factors of interest

**Appendix Table 5.** Country-specific prevalence of concurrence of multiple leading risk factors

**Appendix Table 6.** Prevalence of concurrence of multiple leading risk factors, by region and income level

**Appendix Table 7.** Presence of each possible number of concurrent risk factors by child’s mortality status

**Appendix Table 8.** Chances of under-5 mortality by weighted risk scores

**Appendix Figure 1.** Area under the ROC curve of mutually-adjusted logistic regression model.

**Appendix Figure 2.** Relative ranking of risk factors associated with under-5 mortality from single- adjusted models, pooled analysis of 61 countries

**Appendix Figure 3.** Relative ranking of risk factors associated with neonatal mortality, post-neonatal mortality, infant mortality, and childhood mortality from mutually- adjusted models

**Appendix Figure 4.** Associations between weighted risk score and under-5 mortality, neonatal mortality, post-neonatal mortality, infant mortality, and childhood mortality; odds ratio (ORs) and 95% confidence interval (CI)

**Appendix Figure 5.** Relative ranking of risk factors associated with under-5 mortality from mutually- adjusted models, including household possession of mosquito malaria as risk factor.

**Appendix Figure 6.** Relative ranking of risk factors associated with under-5 mortality from mutually- adjusted models, stratified by sex of children

**Appendix Figure 7.** Association between concurrence of multiple leading risk factors with under-5 mortality, neonatal mortality, post-neonatal mortality, infant mortality, and childhood mortality; odds ratio (ORs) and 95% confidence interval (CI), stratified by income groups

**Appendix Figure 8.** Association between concurrence of multiple leading risk factors with under-5 mortality; odds ratio (ORs) and 95% confidence interval (CI), missing value as separate categories.

**Appendix Figure 9.** Association between concurrence of multiple leading risk factors with under-5 mortality, neonatal mortality, post-neonatal mortality, infant mortality, and childhood mortality; odds ratio (ORs) and 95% confidence interval (CI), stratified by armed conflict events

**Appendix Figure 10.** Association between concurrence of 16 significant risk factors with under-5 mortality, neonatal mortality, post-neonatal mortality, infant mortality, and childhood mortality; odds ratio (ORs) and 95% confidence interval (CI)

**Appendix Figure 11.** Association between concurrence of multiple leading risk factors with under-5 mortality, neonatal mortality, post-neonatal mortality, infant mortality, and childhood mortality; odds ratio (ORs) and 95% confidence interval (CI), 33 countries with observation on all risk factors of interest

**Appendix Figure 12.** Association between concurrence of multiple leading risk factors with under-5 mortality, neonatal mortality, post-neonatal mortality, infant mortality, and childhood mortality; odds ratio (ORs) and 95% confidence interval (CI), No-MI dataset

**Appendix Figure 13.** Association between concurrence of multiple leading risk factors with under-5 mortality, neonatal mortality, post-neonatal mortality, infant mortality, and childhood mortality; odds ratio (ORs) and 95% confidence interval (CI), with adjustment of year-fixed effects

**Appendix Figure 14.** Association between concurrence of multiple leading risk factors with under-5 mortality, neonatal mortality, post-neonatal mortality, infant mortality, and childhood mortality; odds ratio (ORs) and 95% confidence interval (CI), including all children without application of any exclusion criteria (N= 851,255)

**Appendix Table 1. Countries included in the study**

|  | **DHS Sample (N= 61)** | | **Non-DHS Sample (N=73)^a^** | |
| --- | --- | --- | --- | --- |
| **U5MR (Per 1,000)** | Mean (SD) | Range | Mean (SD) | Range |
|  | 43.1 (25.5) | 2-107 | 35.1 (29.9) | 2.8-126 |
| **Included Countries** | **Survey year** | **Income group** | **N** | **U5MR (per 1,000)** |
| Afghanistan | 2015 | Low income | 19,574 | 47 |
| Albania | 2017-2018 | Upper-middle income | 2,320 | 2 |
| Angola | 2015-2016 | Lower-middle income | 8,786 | 41 |
| Armenia | 2015-2016 | Upper-middle income | 1,381 | 4 |
| Bangladesh | 2017-2018 | Lower-middle income | 7,482 | 22 |
| Benin | 2017-2018 | Lower-middle income | 8,744 | 69 |
| Burkina Faso | 2010 | Low income | 10,133 | 91 |
| Burundi | 2016-2017 | Low income | 8,527 | 52 |
| Cambodia | 2014 | Lower-middle income | 5,841 | 21 |
| Cameroon | 2018 | Lower-middle income | 6,302 | 59 |
| Chad | 2014-2015 | Low income | 10,938 | 10 |
| Colombia | 2015 | Upper-middle income | 9,900 | 10 |
| Comoros | 2012 | Lower-middle income | 1,968 | 34 |
| Congo | 2011-2012 | Lower-middle income | 6,312 | 61 |
| Congo Democratic Republic | 2013-2014 | Low income | 11,066 | 100 |
| Cote d'Ivoire | 2011-2012 | Lower-middle income | 5,293 | 72 |
| Dominican Republic | 2013 | Upper-middle income | 2,925 | 28 |
| Egypt | 2014 | Lower-middle income | 11,246 | 17 |
| Ethiopia | 2016 | Low income | 7,082 | 60 |
| Gabon | 2012 | Upper-middle income | 4,047 | 82 |
| Gambia | 2019-2020 | Low income | 5,694 | 39 |
| Ghana | 2014 | Lower-middle income | 4,187 | 47 |
| Guatemala | 2014-2015 | Upper-middle income | 9,451 | 26 |
| Guinea | 2018 | Low income | 5,389 | 87 |
| Haiti | 2016-2017 | Lower-middle income | 4,905 | 61 |
| Honduras | 2011-2012 | Lower-middle income | 8,643 | 22 |
| India | 2019-2021 | Lower-middle income | 175,206 | 29 |
| Indonesia | 2017 | Lower-middle income | 15,237 | 22 |
| Jordan | 2017-2018 | Upper-middle income | 7,156 | 15 |
| Kenya | 2014 | Lower-middle income | 14,713 | 33 |
| Kyrgyz Republic | 2012 | Lower-middle income | 3,113 | 18 |
| Lesotho | 2014 | Lower-middle income | 2,560 | 63 |
| Liberia | 2019-2020 | Low income | 4,180 | 67 |
| Madagascar | 2021 | Low income | 9,216 | 53 |
| Malawi | 2015-2016 | Low income | 13,161 | 39 |
| Maldives | 2017-2018 | Upper-middle income | 2,641 | 15 |
| Mali | 2018 | Low income | 6,250 | 61 |
| Mauritania | 2019-2021 | Lower-middle income | 7,491 | 32 |
| Mozambique | 2011 | Low income | 7,455 | 71 |
| Myanmar | 2015-2016 | Lower-middle income | 3,830 | 32 |
| Namibia | 2013 | Upper-middle income | 3,920 | 51 |
| Nepal | 2016 | Lower-middle income | 3,976 | 21 |
| Niger | 2012 | Lower-middle income | 7,534 | 107 |
| Nigeria | 2018 | Lower-middle income | 21,349 | 94 |
| Pakistan | 2017-2018 | Lower-middle income | 8,159 | 54 |
| Papua New Guinea | 2016-2018 | Lower-middle income | 6,563 | 34 |
| Peru | 2012 | Upper-middle income | 7,931 | 13 |
| Philippines | 2017 | Lower-middle income | 7,923 | 27 |
| Rwanda | 2019-2020 | Low income | 6,062 | 28 |
| Senegal | 2019 | Lower-middle income | 4,250 | 24 |
| Sierra Leone | 2019 | Lower-middle income | 7,202 | 85 |
| South Africa | 2016 | Upper-middle income | 2,993 | 27 |
| Tajikistan | 2017 | Lower-middle income | 4,185 | 22 |
| Tanzania | 2015-2016 | Lower-middle income | 6,912 | 42 |
| Timor-Leste | 2016 | Lower-middle income | 4,867 | 60 |
| Togo | 2013-2014 | Low income | 4,884 | 64 |
| Turkey | 2013 | Upper-middle income | 2,812 | 9 |
| Uganda | 2016 | Low income | 10,064 | 47 |
| Yemen | 2013 | Low income | 10,444 | 39 |
| Zambia | 2018 | Low income | 7,250 | 37 |
| Zimbabwe | 2015 | Lower-middle income | 4,747 | 32 |

**^a^** Data is derived from the 2015 UNICEF on Under-5 Mortality Rate.

**Appendix Table 2. Selection of risk factors**

| **Risk factors** | **Data availability** | **Example References** |
| --- | --- | --- |
| **Household characteristics** | | |
| **Drinking water source** | Data available | Ezeh OK, Agho KE, Dibley MJ, Hall J, Page AN. The impact of water and sanitation on childhood mortality in Nigeria: evidence from demographic and health surveys, 2003–2013. International journal of environmental research and public health. 2014 Sep;11(9):9256-72. |
| **Household Possession of Mosquito Nets** | Data available | Konlan KD, Kossi Vivor N, Gegefe I, Hayford L. Factors associated with ownership and utilization of insecticide treated nets among children under five years in sub-Saharan Africa. BMC Public Health 2022; 22(1): 940. |
| **Household wealth** | Data available | Chao F, You D, Pedersen J, Hug L, Alkema L. National and regional under-5 mortality rate by economic status for low-income and middle-income countries: a systematic assessment. The Lancet Global Health. 2018 May 1;6(5):e535-47. |
| **Indoor air pollution** | Data available | Lwin KS, Nomura S, Swe KT, Gilmour S, Alam MA, Hashizume M. Attributable risk of household solid fuel use and second-hand smoke associated with under-5 mortality in 46 low-and lower-middle-income countries, 2010–2020. International Journal of Hygiene and Environmental Health. 2022 Jun 1;243:113986. |
| **Sanitary facility** | Data available | Ezeh OK, Agho KE, Dibley MJ, Hall J, Page AN. The impact of water and sanitation on childhood mortality in Nigeria: evidence from demographic and health surveys, 2003–2013. International journal of environmental research and public health. 2014 Sep;11(9):9256-72. |
| **Type of residence** | Data available | Kayode, G.A., Adekanmbi, V.T. & Uthman, O.A. Risk factors and a predictive model for under-five mortality in Nigeria: evidence from Nigeria demographic and health survey. BMC Pregnancy Childbirth 12, 10 (2012). Https://doi.org/10.1186/1471-2393-12-10 |
| **Parental characteristics** | | |
| **Birth interval** | Data available | Yigzaw M, Enquselassie F. Birth spacing and risk of child mortality at Kalu district South Wollo Zone of Amhara region, Ethiopia. Ethiopian medical journal. 2010 Apr 1;48(2):105-15. |
| **Child marriage** | For some countries/areas, residents tend to cohabit rather than get married | Hossain MM, Abdulla F, Banik R, Yeasmin S, Rahman A. Child marriage and its association with morbidity and mortality of under-5 years old children in Bangladesh. Plos One. 2022 Feb 9;17(2):e0262927. |
| **Current maternal marital status** | Data available | Yaya S, Bishwajit G, Okonofua F, Uthman OA. Under five mortality patterns and associated maternal risk factors in sub-Saharan Africa: a multi-country analysis. Plos one. 2018 Oct 25;13(10):e0205977. |
| **Maternal age at birth** | Data available | Ayotunde T, Mary O, Melvin AO, Faniyi FF. Maternal age at birth and under-5 mortality in Nigeria. East African Journal of Public Health. 2009 Apr 1;6(1):11-4. |
| **Maternal height** | Data available | Özaltin E, Hill K, Subramanian SV. Association of maternal stature with offspring mortality, underweight, and stunting in low-to middle-income countries. Jama. 2010 Apr 21;303(15):1507-16. |
| **Maternal depression** | Data unavailable | Stewart RC, Ashorn P, Umar E, Dewey KG, Ashorn U, Creed F, Rahman A, Tomenson B, Prado EL, Maleta K. Associations between antenatal depression and neonatal outcomes in Malawi. Maternal & child nutrition. 2019 Apr;15(2):e12709. |
| **Maternal smoking status** | Data available | Caleyachetty R, Tait CA, Kengne AP, Corvalan C, Uauy R, Echouffo-Tcheugui JB. Tobacco use in pregnant women: analysis of data from Demographic and Health Surveys from 54 low-income and middle-income countries. The Lancet Global Health. 2014 Sep 1;2(9):e513-20. |
| **Parental education/occupation** | Data available | Soe K, Holland P, Mateus C. Association between maternal education and childhood mortalities in Myanmar. Asia Pacific Journal of Public Health. 2019 Nov;31(8):689-700. |
| **Maternal health care services received** | | |
| **Distance to health facility** | No data on individual-level | Mahesh Karra, Günther Fink, David Canning, Facility distance and child mortality: a multi-country study of health facility access, service utilization, and child health outcomes, International Journal of Epidemiology, Volume 46, Issue 3, June 2017, Pages 817–826, https://doi.org/10.1093/ije/dyw062 |
| **Family planning need** | Data available | Adedini SA, Odimegwu C, Imasiku EN, Ononokpono DN. Unmet need for family planning: implication for under-five mortality in Nigeria. Journal of health, population, and nutrition. 2015 Mar;33(1):187. |
| **Iron/folic acid supplementation intake during pregnancy** | Over 10 countries have no observations regarding this indicator | Nisar YB, Dibley MJ. Iron/folic acid supplementation during pregnancy prevents neonatal and under-five mortality in Pakistan: propensity score matched sample from two Pakistan Demographic and Health Surveys. |
| **Number of antenatal care visits** | Data available | Oduse S, Zewotir T, North D. The impact of antenatal care on under‐five mortality in Ethiopia: a difference‐in‐differences analysis. BMC pregnancy and childbirth. 2021 Dec;21(1):1-9. |
| **Skilled birth attendants at delivery** | Data available | Singh K, Brodish P, Suchindran C. A regional multilevel analysis: can skilled birth attendants uniformly decrease neonatal mortality?. Maternal and child health journal. 2014 Jan;18:242-9. |
| **Tetanus toxoid injection during pregnancy** | Data available | Blencowe H, Lawn J, Vandelaer J, Roper M, Cousens S. Tetanus toxoid immunization to reduce mortality from neonatal tetanus. International journal of epidemiology. 2010 Apr 1;39(suppl_1):i102-9. |
| **Child characteristics** | | |
| **Birth order** | Data available | Kayode, G.A., Adekanmbi, V.T. & Uthman, O.A. Risk factors and a predictive model for under-five mortality in Nigeria: evidence from Nigeria demographic and health survey. BMC Pregnancy Childbirth 12, 10 (2012). Https://doi.org/10.1186/1471-2393-12-10 |
| **Breastfeeding initiation** | Data available | Smith ER, Hurt L, Chowdhury R, Sinha B, Fawzi W, Edmond KM, et al. Delayed breastfeeding initiation and infant survival: A systematic review and meta-analysis. Plos ONE. 20 17; 12(7):e0180722. |
| **Exclusive breastfeeding** | Data unavailable | Cesar G Victora, Rajiv Bahl, Aluísio J D Barros, Giovanny V A França, Susan Horton, Julia Krasevec, Simon Murch, Mari Jeeva Sankar, Neff Walker, Nigel C Rollins, for The Lancet Breastfeeding Series Group* Breastfeeding in the 21st century: epidemiology, mechanisms, and lifelong effect. Lancet 2016; 387: 475-90 |
| **Full vaccination** | Lack of information on vaccination of dead children | Mark E. Mcgovern, David Canning, Vaccination and All-Cause Child Mortality From 1985 to 2011: Global Evidence From the Demographic and Health Surveys |
| **Sex of child** | Data available | Khan JR, Awan N. A comprehensive analysis on child mortality and its determinants in Bangladesh using frailty models. Archives of Public Health. 2017 Dec;75(1):1-0. |
| **Environmental characteristics** | | |
| **Ambient PM2·5** | Data unavailable | Ortigoza A, Gouveia N, Kephart J, Prado-Galbarro F, Friche AA, Sanchez BN, Diez-Roux AV. Association between ambient PM2· 5 and under-5, infant, and child mortality in Latin America, 2010–15: a longitudinal analysis. The Lancet Planetary Health. 2021 Apr 1;5:S16. |
| **Armed conflict** | Data available | Wagner Z, Heft-Neal S, Bhutta ZA, Black RE, Burke M, Bendavid E. Armed conflict and child mortality in Africa: a geospatial analysis. Lancet 2018; 392(10150): 857-65. |

**Appendix Table 3. Variance inflation factor (VIF) for the risk factors**

| **Variable** | **VIF** |
| --- | --- |
| Birth order | 2.27 |
| Maternal age at birth | 1.59 |
| Maternal education | 1.44 |
| Birth interval | 1.34 |
| Sanitary facility | 1.32 |
| Indoor air pollution | 1.31 |
| Skilled birth attendants at delivery | 1.28 |
| Tetanus toxoid injection during pregnancy | 1.22 |
| Place of residence | 1.21 |
| Drinking water source | 1.20 |
| Iron/folic acid supplementation during pregnancy | 1.19 |
| Number of antenatal care visits | 1.18 |
| Maternal occupation | 1.15 |
| Family planning need | 1.11 |
| Household Possession of Mosquito Nets | 1.05 |
| Current maternal marital status | 1.05 |
| Paternal occupation | 1.04 |
| Birth size | 1.02 |
| Breastfeeding initiation | 1.01 |
| Maternal height | 1.01 |
| Maternal smoking | 1.00 |

**Appendix Table 4. Countries with no observation on one or more risk factors of interest**

| **Variable** | **Missing (%)** | **Countries with no observation** |
| --- | --- | --- |
| Paternal occupation | 34.8 | Peru |
| Maternal height | 34.6 | Afghanistan, Angola, Colombia, Indonesia, Papua New Guinea, Philippines, Senegal, Zambia |
| Maternal occupation | 25.9 | / |
| Household Possession of Mosquito Nets | 25.8 | Albania, Armenia, Bangladesh, Cambodia, Colombia, Dominican Republic, Egypt, Ethiopia, Guatemala, Honduras, Indonesia, Jordan, Kyrgyz Republic, Lesotho, Maldives, Nepal, Peru, Philippines, South Africa, Tajikistan, Turkey, Yemen |
| Maternal smoking | 11.0 | Bangladesh, Chad, Colombia, Egypt, Senegal, Turkey, Yemen |
| Birth size | 7.4 | Bangladesh, Colombia, Maldives, Philippines |
| Number of tetanus injection during pregnancy | 4.9 | Albania, Armenia, Bangladesh, Kyrgyz Republic, Tajikistan, Turkey |
| Sanitary facility | 4.7 | / |
| Current maternal marital status | 4.0 | / |
| Breastfeeding initiation | 3.6 | / |
| Drinking water source | 2.6 | / |
| Indoor air pollution | 2.4 | Egypt, Turkey |
| Number of antenatal care visits | 1.9 | / |
| Family planning need | 1.5 | / |
| Skilled birth attendants at delivery | 0.5 | / |
| Birth order | 0 | / |
| Types of residence | 0 | / |
| Household wealth | 0 | / |
| Maternal occupation | 0 | / |
| Birth interval | 0 | / |
| Maternal age at birth | 0 | / |

**Appendix Table 5. Country-specific prevalence (%) of concurrence of multiple leading risk factors**

|  | **Prevalence (%) and 95% Confidence interval** | | | | | | |
| --- | --- | --- | --- | --- | --- | --- | --- |
|  | **0 risk factor** | **1 risk factor** | **2 risk factors** | **3 risk factors** | **4 risk factors** | **5 risk factors** | **≥ 2 risk factors** |
| Afghanistan | 1.0 (0.9-1.2) | 8.7 (8.3- 9.1) | 32.3 (31.6- 32.9) | 36.9 (36.2- 37.6) | 18.0 (17.5- 18.6) | 3.0 (2.8- 3.3) | 90.3 (89.9-90.7) |
| Albania | 5.9 (5.1- 7.0) | 29.9 (28.0- 31.8) | 41.2 (39.2- 43.2) | 20.8 (19.2- 22.5) | 2.1 (1.6- 2.8) | 0.1 (0.0- 0.3) | 64.2 (62.2-66.1) |
| Angola | 2.7 (2.4- 3.0) | 19.0 (18.2- 19.8) | 38.0 (37.0- 39.0) | 29.8 (28.8- 30.7) | 9.3 (8.7- 9.9) | 1.3 (1.0- 1.5) | 78.3 (77.4-79.2) |
| Armenia | 11.3 (9.7- 13.1) | 33.4 (30.9- 35.9) | 37.1 (34.6- 39.7) | 16.3 (14.4- 18.3) | 2.0 (1.3- 2.8) | / | 55.3 (52.7-57.9) |
| Bangladesh | / | 14.7 (13.9- 15.5) | 44.6 (43.5- 45.8) | 31.4 (30.4- 32.5) | 8.9 (8.3- 9.6) | 0.3 (0.2- 0.5) | 82.3 (84.5-86.1) |
| Benin | 2.1 (1.8- 2.4) | 13.6 (12.9- 14.4) | 37.5 (36.5- 38.6) | 34.7 (33.8- 35.7) | 10.9 (10.2- 11.5) | 1.1 (0.9- 1.4) | 84.3 (83.5-85.0) |
| Burkina Faso | 1.5 (1.3- 1.8) | 8.5 (8.0- 9.1) | 32.6 (31.7- 33.5) | 40.6 (39.6- 41.5) | 15.1 (14.4- 15.8) | 1.6 (1.4- 1.9) | 90.0 (89.3-90.5) |
| Burundi | 8.3 (7.8- 8.9) | 30.7 (29.8- 31.7) | 39.8 (38.7- 40.8) | 17.2 (16.4- 18.0) | 3.6 (3.2- 4.0) | 0.3 (0.2- 0.4) | 60.1 (60.0-61.9) |
| Cambodia | 14.5 (13.6- 15.4) | 32.6 (31.5- 33.9) | 33.2 (32.0- 34.4) | 16.1 (15.2- 17.1) | 3.2 (2.8- 3.7) | 0.3 (0.2- 0.5) | 52.9 (51.6-54.2) |
| Cameroon | 6.6 (6.0- 7.2) | 24.4 (23.3- 25.5) | 35.8 (34.6- 37.0) | 25.9 (24.9- 27.0) | 6.6 (6.1- 7.3) | 0.7 (0.5- 0.9) | 69.1 (67.9-70.2) |
| Chad | 0.5 (0.4- 0.7) | 4.7 (4.3- 5.1) | 24.8 (24.0- 25.6) | 43.7 (42.8- 44.6) | 22.5 (21.7- 23.3) | 3.8 (3.5- 4.2) | 94.8 (94.3-95.2) |
| Colombia | / | 24.2 (23.4- 25.0) | 58.6 (57.6- 59.5) | 16.8 (16.1- 17.6) | 0.4 (0.3- 0.6) | / | 75.8 (75.0-76.6) |
| Comoros | 2.9 (2.2- 3.7) | 14.3 (12.8- 15.9) | 31.6 (29.6- 33.7) | 34.2 (32.2- 36.4) | 14.7 (13.2- 16.3) | 2.2 (1.7- 3.0) | 82.8 (81.0-84.4) |
| Congo | 6.3 (5.7- 6.9) | 26.2 (25.1- 27.3) | 40.9 (39.7- 42.1) | 22.3 (21.3- 23.3) | 4.1 (3.7- 4.7) | 0.2 (0.1- 0.3) | 67.5 (66.4-68.7) |
| Congo Democratic Republic | 5.1 (4.7- 5.5) | 27.6 (26.8- 28.5) | 38.8 (37.9- 39.7) | 22.9 (22.1- 23.7) | 5.2 (4.8- 5.6) | 0.5 (0.4- 0.6) | 67.3 (66.4-68.2) |
| Cote d'Ivoire | 1.3 (1.0- 1.6) | 8.9 (8.2- 9.7) | 28.3 (27.1- 29.6) | 39.3 (38.0- 40.6) | 19.6 (18.6- 20.7) | 2.6 (2.2- 3.1) | 89.8 (89.0-90.6) |
| Dominican Republic | 10.4 (9.3- 11.6) | 32.2 (30.5- 33.9) | 35.6 (33.8- 37.3) | 18.0 (16.7- 19.5) | 3.8 (3.2- 4.6) | 0.0 (0.0- 0.2) | 57.4 (55.6-59.2) |
| Egypt | 7.9 (7.4- 8.4) | 29.4 (28.6- 30.3) | 35.4 (34.5- 36.3) | 21.7 (21.0- 22.5) | 5.1 (4.7- 5.5) | 0.5 (0.4- 0.6) | 62.7 (61.8-63.6) |
| Ethiopia | 4.0 (3.6- 4.5) | 19.4 (18.5- 20.3) | 35.6 (34.5- 36.7) | 27.5 (26.5- 28.5) | 11.7 (10.9- 12.4) | 1.9 (1.6- 2.2) | 76.7 (75.7-77.6) |
| Gabon | 5.1 (4.5- 5.9) | 22.4 (21.2- 23.7) | 40.4 (38.9- 41.9) | 26.9 (25.5- 28.3) | 4.9 (4.3- 5.7) | 0.2 (0.1- 0.4) | 72.4 (71.0-73.8) |
| Gambia | 2.6 (2.2- 3.0) | 15.8 (14.9- 16.8) | 35.3 (34.1- 36.5) | 32.7 (31.5- 34.0) | 12.1 (11.3- 13.0) | 1.5 (1.2- 1.8) | 81.6 (80.6-82.6) |
| Ghana | 6.2 (5.5- 6.9) | 23.1 (21.9- 24.4) | 35.0 (33.6- 36.5) | 26.9 (25.5- 28.2) | 8.3 (7.5- 9.1) | 0.5 (0.4- 0.8) | 70.7 (69.3-72.1) |
| Guatemala | 11.3 (10.6- 11.9) | 32.3 (31.4- 33.2) | 35.3 (34.3- 36.2) | 17.4 (16.6- 18.2) | 3.5 (3.1- 3.9) | 0.3 (0.2- 0.4) | 56.4 (55.4-57.4) |
| Guinea | 1.2 (0.9- 1.5) | 9.2 (8.5- 10.0) | 28.7 (27.5- 29.9) | 41.5 (40.2- 42.9) | 17.5 (16.5- 18.5) | 1.9 (1.5- 2.3) | 89.6 (88.7-90.3) |
| Haiti | 4.9 (4.3- 5.6) | 22.1 (21.0- 23.3) | 35.6 (34.3- 37.0) | 28.0 (26.7- 29.2) | 8.6 (7.8- 9.4) | 0.8 (0.6- 1.1) | 73.0 (71.7-74.2) |
| Honduras | 13.6 (12.9- 14.3) | 34.8 (33.9- 35.9) | 34.0 (33.0- 35.0) | 14.9 (14.2- 15.7) | 2.5 (2.2- 2.9) | 0.2 (0.1- 0.3) | 51.6 (50.5-52.6) |
| India | 9.8 (9.7- 9.9) | 29.4 (29.2- 29.6) | 35.4 (35.2- 35.7) | 20.6 (20.4- 20.8) | 4.4 (4.3- 4.5) | 0.4 (0.3- 0.4) | 60.8 (60.6-61.0) |
| Indonesia | 11.5 (11.0- 12.0) | 37.0 (36.3- 37.8) | 35.7 (34.9- 36.4) | 13.8 (13.2- 14.3) | 2.0 (1.8- 2.3) | 0.0 (0.0- 0.1) | 51.5 (50.7-52.3) |
| Jordan | 19.7 (18.8- 20.7) | 37.8 (36.7- 39.0) | 29.4 (28.4- 30.5) | 11.2 (10.4- 11.9) | 1.8 (1.5- 2.2) | 0.0 (0.0- 0.1) | 42.4 (41.3-43.6) |
| Kenya | 7.0 (6.6- 7.4) | 15.5 (14.9- 16.1) | 37.5 (36.7- 38.3) | 35.8 (35.0- 36.5) | 4.1 (3.8- 4.4) | 0.2 (0.1- 0.3) | 77.5 (76.9-78.2) |
| Kyrgyz Republic | 13.8 (12.6- 15.1) | 39.8 (38.1- 41.5) | 34.8 (33.1- 36.4) | 9.9 (8.9- 11.0) | 1.7 (1.3- 2.2) | / | 46.4 (44.6-48.1) |
| Lesotho | 14.9 (13.6- 16.4) | 37.1 (35.3- 39.0) | 33.8 (32.0- 35.6) | 12.0 (10.8- 13.4) | 2.1 (1.6- 2.7) | 0.0 (0.0- 0.3) | 47.9 (46.0-49.9) |
| Liberia | 6.2 (5.5- 7.0) | 24.2 (22.9- 25.5) | 37.8 (36.3- 39.2) | 24.4 (23.1- 25.7) | 6.9 (6.2- 7.7) | 0.6 (0.4- 0.8) | 69.6 (68.2-70.9) |
| Madagascar | 8.0 (7.4- 8.5) | 28.0 (27.1- 29.0) | 34.1 (33.1- 35.1) | 22.0 (21.1- 22.8) | 6.9 (6.4- 7.4) | 1.1 (0.9- 1.3) | 94.0 (63.0-65.0) |
| Malawi | 21.7 (21.0- 22.5) | 37.3 (36.5- 38.1) | 29.0 (28.2- 29.8) | 10.1 (9.6- 10.7) | 1.8 (1.6- 2.0) | 0.1 (0.0- 0.1) | 41.0 (40.1-41.8) |
| Maldives | / | 5.5 (4.6- 6.4) | 28.9 (27.2- 30.7) | 46.9 (45.0- 48.8) | 18.6 (17.1- 20.1) | 0.2 (0.1- 0.4) | 94.5 (93.6-95.4) |
| Mali | 2.6 (2.3- 3.1) | 13.8 (12.9- 14.7) | 35.7 (34.5- 36.9) | 33.9 (32.7- 35.1) | 12.0 (11.2- 12.8) | 2.0 (1.7- 2.4) | 83.6 (82.7-84.5) |
| Mauritania | 2.5 (2.1- 2.8) | 16.2 (15.4- 17.1) | 33.1 (32.1- 34.2) | 31.8 (30.8- 32.9) | 14.1 (13.3- 14.9) | 2.3 (2.0- 2.7) | 81.3 (90.4-92.2) |
| Mozambique | 4.8 (4.4- 5.3) | 27.2 (26.2- 28.3) | 41.0 (39.9- 42.1) | 21.8 (20.9- 22.7) | 4.7 (4.2- 5.2) | 0.5 (0.3- 0.6) | 67.9 (66.9-69.0) |
| Myanmar | 10.7 (9.8- 11.7) | 34.3 (32.8- 35.8) | 34.8 (33.3- 36.3) | 15.9 (14.8- 17.1) | 3.9 (3.3- 4.6) | 0.4 (0.3- 0.7) | 55.0 (53.5-56.6) |
| Namibia | 13.9 (12.9- 15.0) | 36.2 (34.8- 37.8) | 34.1 (32.6- 35.6) | 13.0 (12.0- 14.1) | 2.6 (2.2- 3.2) | 0.1 (0.1- 0.3) | 49.8 (48.3-51.4) |
| Nepal | 6.2 (5.5- 7.0) | 23.2 (21.9- 24.5) | 38.1 (36.6- 39.6) | 24.9 (23.6- 26.3) | 6.7 (6.0- 7.5) | 0.9 (0.7- 1.3) | 70.6 (69.2-72.0) |
| Niger | 2.3 (2.0- 2.7) | 12.3 (11.5- 13.0) | 31.3 (30.3- 32.4) | 36.5 (35.4- 37.6) | 15.2 (14.4- 16.0) | 2.4 (2.0- 2.7) | 85.4 (84.6-86.2) |
| Nigeria | 4.5 (4.2- 4.8) | 18.2 (17.7- 18.7) | 32.8 (32.1- 33.4) | 31.9 (31.3- 32.5) | 11.5 (11.0- 11.9) | 1.2 (1.0- 1.3) | 77.3 (76.7-77.8) |
| Pakistan | 3.1 (2.7- 3.5) | 16.2 (15.4- 17.0) | 30.8 (29.8- 31.8) | 32.8 (31.8- 33.9) | 14.7 (14.0- 15.5) | 2.4 (2.1- 2.8) | 80.7 (79.9-81.6) |
| Papua New Guinea | 10.4 (9.7- 11.2) | 28.5 (27.4- 29.6) | 33.7 (32.6- 34.9) | 20.8 (19.8- 21.8) | 5.9 (5.4- 6.5) | 0.7 (0.5- 0.9) | 61.1 (59.9-62.3) |
| Peru | 12.8 (12.1- 13.6) | 35.2 (34.2- 36.3) | 35.6 (34.6- 36.7) | 14.0 (13.2- 14.8) | 2.3 (2.0- 2.7) | / | 51.9 (50.8-53.0) |
| Philippines | / | 17.2 (16.4- 18.1) | 38.3 (37.2- 39.4) | 32.7 (31.7- 33.7) | 11.5 (10.9- 12.3) | 0.2 (0.1- 0.4) | 82.8 (81.9-83.6) |
| Rwanda | 23.9 (22.8- 24.9) | 39.6 (38.3- 40.8) | 26.8 (25.7- 27.9) | 8.4 (7.7- 9.1) | 1.4 (1.1- 1.7) | 0.0 (0.0- 0.1) | 36.6 (35.4-37.8) |
| Senegal | 2.4 (2.0- 2.9) | 11.7 (10.7- 12.7) | 28.5 (27.2- 29.9) | 34.2 (32.8- 35.6) | 18.6 (17.5- 19.8) | 4.6 (4.0- 5.2) | 85.9 (84.9-86.9) |
| Sierra Leone | 4.4 (3.9- 4.9) | 20.1 (19.2- 21.0) | 40.4 (39.3- 41.5) | 26.9 (25.8- 27.9) | 7.4 (6.9- 8.1) | 0.8 (0.7- 1.1) | 75.5 (74.5-76.5) |
| South Africa | 12.3 (11.2- 13.5) | 38.1 (36.4- 39.8) | 34.8 (33.1- 36.5) | 13.0 (11.8- 14.2) | 1.8 (1.4- 2.4) | 0.0 (0.0- 0.2) | 49.6 (47.8-51.4) |
| Tajikistan | 8.6 (7.8- 9.5) | 31.9 (30.5- 33.4) | 37.3 (35.9- 38.8) | 19.6 (18.4- 20.8) | 2.5 (2.1- 3.0) | / | 59.5 (58.0-60.9) |
| Tanzania | 9.4 (8.7- 10.1) | 28.6 (27.6- 29.7) | 36.8 (35.7- 37.9) | 20.3 (19.4- 21.3) | 4.4 (4.0- 4.9) | 0.5 (0.3- 0.7) | 62.0 (60.8-63.1) |
| Timor Leste | 8.4 (7.7- 9.3) | 29.1 (27.8- 30.3) | 34.9 (33.6- 36.3) | 21.6 (20.5- 22.8) | 5.2 (4.6- 5.8) | 0.8 (0.6- 1.0) | 62.5 (61.1-63.9) |
| Togo | 4.6 (4.1- 5.3) | 21.0 (19.9- 22.2) | 36.8 (35.4- 38.1) | 27.2 (26.0- 28.5) | 9.4 (8.6- 10.2) | 1.1 (0.8- 1.4) | 74.4 (73.1-75.6) |
| Turkey | 15.4 (14.1- 16.7) | 35.2 (33.5- 37.0) | 31.2 (29.5- 32.9) | 14.0 (12.8- 15.4) | 3.6 (3.0- 4.4) | 0.6 (0.4- 1.0) | 49.4 (47.6-51.3) |
| Uganda | 12.6 (12.0- 13.3) | 32.2 (31.3- 33.1) | 34.3 (33.4- 35.3) | 16.9 (16.2- 17.7) | 3.7 (3.4- 4.1) | 0.2 (0.1- 0.3) | 55.1 (54.2-56.1) |
| Yemen | 5.8 (5.4- 6.3) | 18.5 (17.8- 19.3) | 32.3 (31.4- 33.2) | 28.0 (27.1- 28.9) | 12.8 (12.2- 13.5) | 2.6 (2.3- 2.9) | 75.7 (74.8-76.5) |
| Zambia | 18.8 (17.9- 19.7) | 36.8 (35.7- 37.9) | 30.7 (29.7- 31.8) | 11.7 (11.0- 12.5) | 1.9 (1.6- 2.2) | 0.1 (0.1- 0.3) | 44.4 (43.4-45.6) |
| Zimbabwe | 19.5 (18.4- 20.7) | 38.8 (37.4- 40.2) | 30.2 (28.9- 31.5) | 10.4 (9.5- 11.3) | 1.1 (0.9- 1.5) | / | 41.7 (40.3-43.1) |

* The concurrence of multiple risk factors was computed by counting the presence of the top 5 risk factors (i.e., unsatisfied family planning, short birth interval (<18 months), small birth size, never breastfed or delayed breastfeeding, and low maternal education (no schooling)) for each individual.

**Appendix Table 6. Prevalence (%) of concurrence of multiple leading risk factors, by region and income level.**

|  | **Prevalence (%) and 95% CI** | | | | | | |
| --- | --- | --- | --- | --- | --- | --- | --- |
|  | **0 risk factor** | **1 risk factor** | **2 risk factors** | **3 risk factors** | **4 risk factors** | **5 risk factors** | **≥ 2 risk factors** |
| **By region** | | | | | | | |
| East Asia and Pacific | 9.3 (9.0- 9.5) | 30.5 (30.1- 31.0) | 35.4 (34.9- 35.8) | 19.6 (19.2- 19.9) | 5.0 (4.8- 5.2) | 0.3 (0.3- 0.4) | 60.2 (59.8-60.7) |
| Europe and central Asia | 11.0 (10.5- 11.5) | 34.2 (33.4- 35.0) | 36.1 (35.3- 36.9) | 16.2 (15.5- 16.8) | 2.4 (2.2- 2.7) | 0.1 (0.1- 0.2) | 54.8 (54.0-55.7) |
| Latin America and the Caribbean | 8.7 (8.4- 8.9) | 30.4 (29.9- 30.8) | 40.4 (39.9- 40.9) | 17.4 (17.0- 17.7) | 3.0 (2.8- 3.1) | 0.2 (0.2- 0.2) | 61.0 (60.5-61.4) |
| Middle east and north Africa | 10.1 (9.7- 10.4) | 27.6 (27.0- 28.1) | 32.8 (32.2- 33.3) | 21.4 (20.9- 21.8) | 7.1 (6.8- 7.4) | 1.1 (1.0- 1.3) | 62.4 (61.8-62.9) |
| South Asia | 8.2 (8.1- 8.3) | 26.1 (25.9- 26.3) | 35.3 (35.1- 35.5) | 23.3 (23.1- 23.5) | 6.4 (6.3- 6.5) | 0.7 (0.7- 0.7) | 65.7 (65.5-65.9) |
| Sub-Saharan Africa | 7.1 (7.0- 7.2) | 22.1 (22.0- 22.3) | 34.3 (34.1- 34.5) | 26.8 (26.6- 27.0) | 8.6 (8.5- 8.7) | 1.1 (1.0- 1.1) | 70.8 (70.6-71.0) |
| **By income level** | | | | | | | |
| Low income | 7.3 (7.2- 7.4) | 21.7 (21.5- 21.9) | 33.3 (33.1- 33.5) | 26.4 (26.2- 26.7) | 9.8 (9.7- 10.0) | 1.4 (1.4- 1.5) | 71.0 (70.8-71.2) |
| Lower-middle income | 8.0 (7.9- 8.1) | 26.0 (25.8- 26.1) | 35.4 (35.2- 35.5) | 23.7 (23.6- 23.8) | 6.3 (6.2- 6.4) | 0.6 (0.6- 0.7) | 66.0 (65.9-66.2) |
| Upper-middle income | 9.8 (9.6- 10.1) | 30.7 (30.3- 31.1) | 38.7 (38.3- 39.1) | 17.5 (17.2- 17.8) | 3.2 (3.0- 3.3) | 0.1 (0.1- 0.2) | 59.5 (59.1-59.9) |

* The concurrence of multiple risk factors was computed by counting the presence of the top 5 risk factors (i.e., unsatisfied family planning, short birth interval (<18 months), small birth size, never breastfed or delayed breastfeeding, and low maternal education (no schooling)) for each individual.

**Appendix Table 7. Presence of each possible number of concurrent risk factors by child’s mortality status**

|  | **All children, No.** | **Dead children, No.** | **Percentage** |
| --- | --- | --- | --- |
| **Overall** | | | |
| Total | 604,372 | 18,166 | 3.0% (2.9%-3.1%) |
| 0 risk factor | 48,302(8.0%) | 522(2.9%) | 1.1% (1.0%-1.2%) |
| 1 risk factor | 152,945(25.3%) | 2,541(14.0%) | 1.7% (1.6%-1.7%) |
| 2 risk factors | 212,407(35.2%) | 5,615(30.9%) | 2.6% (2.6%-2.7%) |
| 3 risk factors | 144,014(23.8%) | 6,243(34.4%) | 4.3% (4.2%-4.4%) |
| 4 risk factors | 41,903(6.9%) | 2,800(15.4%) | 6.7% (6.4%-6.9%) |
| 5 risk factors | 4,801(0.8%) | 445(2.5%) | 9.3% (8.4%-10.1%) |
| *≥ 2 risk factors* | *403,125 (66.7%)* | *15,103 (83.1%)* | *3.7% (3.7%-3.8%)* |
| **By Regions** | | | |
| ***East Asia and Pacific*** | | | |
| Total | 44,261 | 957 | 2.2% (2.0%-2.3%) |
| 0 risk factor | 4,100(9.3%) | 33(3.5%) | 0.8% (0.5%-1.1%) |
| 1 risk factor | 13,510(30.5%) | 145(15.2%) | 1.1% (0.9%-1.2%) |
| 2 risk factors | 15,654(35.3%) | 326(34.1%) | 2.1% (1.9%-2.3%) |
| 3 risk factors | 8,657(19.6%) | 302(31.6%) | 3.5% (3.1%-3.9%) |
| 4 risk factors | 2,200(5.0%) | 141(14.6%) | 6.4% (5.4%-7.4%) |
| 5 risk factors | 140(0.3%) | 10(1.0%) | 7.1% (2.9%-11.4%) |
| *≥ 2 risk factors* | *26,651 (60.2%)* | *779 (81.4%)* | *2.9% (2.7%-3.1%)* |
| ***Europe and Central Asia*** | | | |
| Total | 13,811 | 154 | 1.1% (0.9%-1.3%) |
| 0 risk factor | 1,517(11.0%) | 7(4.6%) | 0.5% (0.1%-0.8%) |
| 1 risk factor | 4,719(34.1%) | 23(14.9%) | 0.5% (0.3%-0.7%) |
| 2 risk factors | 4,989(36.1%) | 58(37.7%) | 1.2% (0.9%-1.5%) |
| 3 risk factors | 2,231(16.2%) | 48(31.2%) | 2.2% (1.5%-2.8%) |
| 4 risk factors | 336(2.4%) | 18(11.7%) | 5.4% (2.9%-7.8%) |
| 5 risk factors | 19(0.2%) | / | / |
| *≥ 2 risk factors* | *7,575 (54.9%)* | *124 (80.5%)* | *1.6% (1.4%-1.9%)* |
| ***Latin America and the Caribbean*** | | | |
| Total | 43,755 | 847 | 1.9% (1.8%-2.1%) |
| 0 risk factor | 3,797(8.7%) | 33 (3.9%) | 0.9% (0.6%-1.2%) |
| 1 risk factor | 13,280(30.4%) | 185(21.8%) | 1.4% (1.2%-1.6%) |
| 2 risk factors | 17,680(40.4%) | 306(36.1%) | 1.7% (1.5%-1.9%) |
| 3 risk factors | 7,607(17.4%) | 232(27.4%) | 3.0% (2.7%-3.4%) |
| 4 risk factors | 1,305(2.9%) | 82(9.7%) | 6.3% (5.0%-7.6%) |
| 5 risk factors | 86(0.2%) | 9(1.1%) | 10.5% (4.0%-16.9%) |
| *≥ 2 risk factors* | *26,678 (61.0%)* | *629 (74.3%)* | *2.4% (2.2%-2.5%)* |
| ***Middle East and North Africa*** | | | |
| Total | 28,846 | 566 | 2.0% (1.8%-2.1%) |
| 0 risk factor | 2,908(10.0%) | 14(2.5%) | 0.5% (0.2%-0.7%) |
| 1 risk factor | 7,949(27.6%) | 68(12.0%) | 0.9% (0.7%-1.1%) |
| 2 risk factors | 9,456(32.8%) | 152(26.9%) | 1.6% (1.4%-1.9%) |
| 3 risk factors | 6,164(21.4%) | 193(34.1%) | 3.1% (2.7%-3.6%) |
| 4 risk factors | 2,044(7.1%) | 115(20.3%) | 5.6% (4.6%-6.6%) |
| 5 risk factors | 325(1.1%) | 24(4.2%) | 7.4% (4.5%-10.2%) |
| *≥ 2 risk factors* | *17,989 (62.4%)* | *484 (85.5%)* | *2.7% (2.5%-2.9%)* |
| ***South Asia*** | | | |
| Total | 217,038 | 5,526 | 2.5% (2.5%-2.6%) |
| 0 risk factor | 17,854(8.2%) | 141(2.6%) | 0.8% (0.7%-0.9%) |
| 1 risk factor | 56,678(26.1%) | 729(13.2%) | 1.3% (1.2%-1.4%) |
| 2 risk factors | 76,540(35.3%) | 1,607(29.1%) | 2.1% (2.0%-2.2%) |
| 3 risk factors | 50,552(23.3%) | 1,993(36.1%) | 3.9% (3.8%-4.1%) |
| 4 risk factors | 13,927(6.4%) | 910(16.5%) | 6.5% (6.1%-6.9%) |
| 5 risk factors | 1,487(0.7%) | 146(2.5%) | 9.8% (8.3%-11.3%) |
| *≥ 2 risk factors* | *142,506 (65.7%)* | *4,656 (84.3%)* | *3.3% (3.2%-3.4%)* |
| ***Sub-Saharan Africa*** | | | |
| Total | 256,661 | 10,116 | 3.9% (3.9%-4.0%) |
| 0 risk factor | 18,126(7.1%) | 294(2.9%) | 1.6% (1.4%-1.8%) |
| 1 risk factor | 56,809(22.1%) | 1,391(13.8%) | 2.4% (2.3%-2.6%) |
| 2 risk factors | 88,088(34.3%) | 3,166(31.3%) | 3.6% (3.5%-3.7%) |
| 3 risk factors | 68,803(26.8%) | 3,475(34.3%) | 5.1% (4.9%-5.2%) |
| 4 risk factors | 22,091(8.6%) | 1,534(15.2%) | 6.9% (6.6%-7.3%) |
| 5 risk factors | 2,744(1.1%) | 256(2.5%) | 9.3% (8.2%-10.4%) |
| *≥ 2 risk factors* | *181,726 (70.8%)* | *8,431 (83.3%)* | *4.6% (4.5%-4.7%)* |
| **By income** | | | |
| ***Low-income countries*** | | | |
| Total | 157,369 | 6,195 | 3.9% (3.8%-4.0%) |
| 0 risk factor | 11,467(7.3%) | 166(2.7%) | 1.4% (1.2%-1.7%) |
| 1 risk factor | 34,193(21.7%) | 835(13.5%) | 2.4% (2.3%-2.6%) |
| 2 risk factors | 52,395(33.3%) | 1,841(29.6%) | 3.5% (3.4%-3.7%) |
| 3 risk factors | 41,600(26.4%) | 2,141(34.6%) | 5.1% (4.9%-5.4%) |
| 4 risk factors | 15,498(9.9%) | 1,028(16.6%) | 6.6% (6.2%-7.0%) |
| 5 risk factors | 2,216(1.4%) | 184(3.0%) | 8.3% (7.2%-9.5%) |
| *≥ 2 risk factors* | *111,709 (71.0%)* | *5,194 (83.8%)* | *4.6% (4.5%-4.8%)* |
| ***Lower-middle income countries*** | | | |
| Total | 389,526 | 10,996 | 2.8% (2.8%-2.9%) |
| 0 risk factor | 31,192(8.0%) | 306(2.8%) | 1.0% (0.9%-1.1%) |
| 1 risk factor | 101104(26.0%) | 1,504(13.7%) | 1.5% (1.4%-1.6%) |
| 2 risk factors | 137,792(35.3%) | 3,423(31.1%) | 2.5% (2.4%-2.6%) |
| 3 risk factors | 92,342(23.7%) | 3,844(35.0%) | 4.2% (4.0%-4.3%) |
| 4 risk factors | 24,580(6.3%) | 1,664(15.1%) | 6.8% (6.5%-7.1%) |
| 5 risk factors | 2,516(0.7%) | 255(2.3%) | 10.1% (9.0%-11.3%) |
| *≥ 2 risk factors* | *257,230 (66.0%)* | *9,186 (83.5%)* | *3.6% (3.5%-3.6%)* |
| ***Upper-middle income countries*** | | | |
| Total | 57,477 | 975 | 1.7% (1.6%-1.8%) |
| 0 risk factor | 5,643(9.8%) | 50(5.1%) | 0.9% (0.6%-1.1%) |
| 1 risk factor | 17,648(30.7%) | 202(20.7%) | 1.1% (1.0%-1.3%) |
| 2 risk factors | 22,220(38.7%) | 351(36.0%) | 1.6% (1.4%-1.7%) |
| 3 risk factors | 10,072(17.5%) | 258(26.5%) | 2.6% (2.3%-2.9%) |
| 4 risk factors | 1,825(3.2%) | 108(11.1%) | 5.9% (4.8%-7.0%) |
| 5 risk factors | 69(0.1%) | 6(0.6%) | 8.7% (2.0%-15.3%) |
| *≥ 2 risk factors* | *34,186 (59.5%)* | 723 (74.2%) | *2.1% (2.0%-2.3%)* |

* The concurrence of multiple risk factors was computed by counting the presence of the top 5 risk factors (i.e., unsatisfied family planning, short birth interval (<18 months), small birth size, never breastfed or delayed breastfeeding, and low maternal education (no schooling)) for each individual.

**Appendix Table 8. Presence of weighted risk score by child’s mortality status**

|  | All children, No. | Dead children, No. | Percentage |
| --- | --- | --- | --- |
| Total | 604,372 | 18,166 | 3.0% (2.9%-3.1%) |
| 0 | 48,123(8.0%) | 511 (2.8%) | 1.1% (1.0%-1.2%) |
| 1~2, not including 2 | 106,853(17.7%) | 1,470 (8.1%) | 1.4% (1.3%-1.4%) |
| 2~4, not including 4 | 244,251(40.4%) | 6,331(34.9%) | 2.6% (2.5%-2.7%) |
| 4~6, not including 6 | 137,309(22.7%) | 5,986 (33.0%) | 4.4% (4.3%-4.5%) |
| >=6 | 67,836 (11.2%) | 3,868 (21.3%) | 5.7% (5.5%-5.9%) |

* The weighted risk scores were calculated on the basis of magnitude β coefficient of the top 5 risk factors (i.e., unsatisfied family planning, short birth interval (<18 months), small birth size, never breastfed or delayed breastfeeding, and low maternal education (no schooling))for each individual.

**Appendix Figure 1. Area under the ROC curve of mutually-adjusted logistic regression model.**

**Appendix Figure 2. Relative ranking of risk factors associated with under-5 mortality from single- adjusted models, pooled analysis of 61 countries (N=604,372)**

* Child sex, child age, and country-fixed effects were adjusted in this model

* Short birth interval – birth interval <18m; Low maternal education – mothers with no education; ANC less than 4 times – antenatal care visit less than 4 times; without IFA supplementary during pregnancy – without iron/folic acid supplementary during pregnancy.

**Appendix Figure 3. Relative ranking of risk factors associated with neonatal mortality, post-neonatal mortality, infant mortality, and childhood mortality from mutually- adjusted models**

1. Neonatal mortality (deaths from birth to 28 days)

* Child sex, child age, and country-fixed effects were adjusted in this model

* Short birth interval – birth interval <18m; Low maternal education – mothers with no education; ANC less than 4 times – antenatal care visit less than 4 times.

1. Post-neonatal mortality (deaths from 29 to 364 days)

* Child sex, child age, and country-fixed effects were adjusted in this model

* Short birth interval – birth interval <18m; Low maternal education – mothers with no education; ANC less than 4 times – antenatal care visit less than 4 times

1. Infant mortality (deaths from 0 to 364 days)

* Child sex, child age, and country-fixed effects were adjusted in this model

* Short birth interval – birth interval <18m; Low maternal education – mothers with no education; ANC less than 4 times – antenatal care visit less than 4 times

1. Childhood mortality (deaths from 365 days to 59 months)

* Child sex, child age, and country-fixed effects were adjusted in this model

* Short birth interval – birth interval <18m; Low maternal education – mothers with no education; ANC less than 4 times – antenatal care visit less than 4 times

**Appendix Figure 4. Associations between weighted risk score and under-5 mortality, neonatal mortality, post-neonatal mortality, infant mortality, and childhood mortality; odds ratio (ORs) and 95% confidence interval (CI)**

**^*^**The weighted risk score was calculated on the basis of magnitude β coefficient of the top 5 risk factors (i.e., unsatisfied family planning, short birth interval (<18 months), small birth size, never breastfed or delayed breastfeeding, and low maternal education (no schooling)).

^*^Neonatal mortality – deaths from 0 to 28 days, post-neonatal mortality – deaths from 29 to 364 days, infant mortality – deaths from 0 to 364 days, childhood mortality – deaths from 365 days to 59 months.

**Appendix Figure 5. Relative ranking of risk factors associated with under-5 mortality from mutually- adjusted models, including household possession of mosquito malaria as risk factor.**

*Based on World Bank data, we choose the following countries with malaria endemic for this analysis: Afghanistan, Angola, Benin, Burkina Faso, Burundi, Cameroon, Chad, Colombia, Comoros, Congo Rep, Congo Democratic Republic, Cote d'Ivoire, Ghana, Guinea, Gabon, Gambia, Haiti, India, Liberia, Madagascar, Malawi, Mali, Kenya, Mauritania, Myanmar, Mozambique, Namibia, Niger, Nigeria, Pakistan, Papua New Guinea, Rwanda, Senegal, Tanzania, Zambia, Zimbabwe, Sierra Leone, Togo, Uganda.

* Child sex, child age, and country-fixed effects were adjusted in this model

* Short birth interval – birth interval <18m; Low maternal education – mothers with no education; ANC less than 4 times – antenatal care visit less than 4 times

**Appendix Figure 6 (1-2). Relative ranking of risk factors associated with under-5 mortality from mutually- adjusted models, stratified by sex of children**

1. **Male (N =** **314,218)**

* Child age, and country-fixed effects were adjusted in this model

* Short birth interval – birth interval <18m; Low maternal education – mothers with no education; ANC less than 4 times – antenatal care visit less than 4 times

1. **Female (N = 290,154)**

* Child age and country-fixed effects were adjusted in this model

* Short birth interval – birth interval <18m; Low maternal education – mothers with no education; ANC less than 4 times – antenatal care visit less than 4 times

**Appendix Figure 7. Association between concurrence of multiple leading risk factors with under-5 mortality, neonatal mortality, post-neonatal mortality, infant mortality, and childhood mortality; odds ratio (ORs) and 95% confidence interval (CI), stratified by income groups**
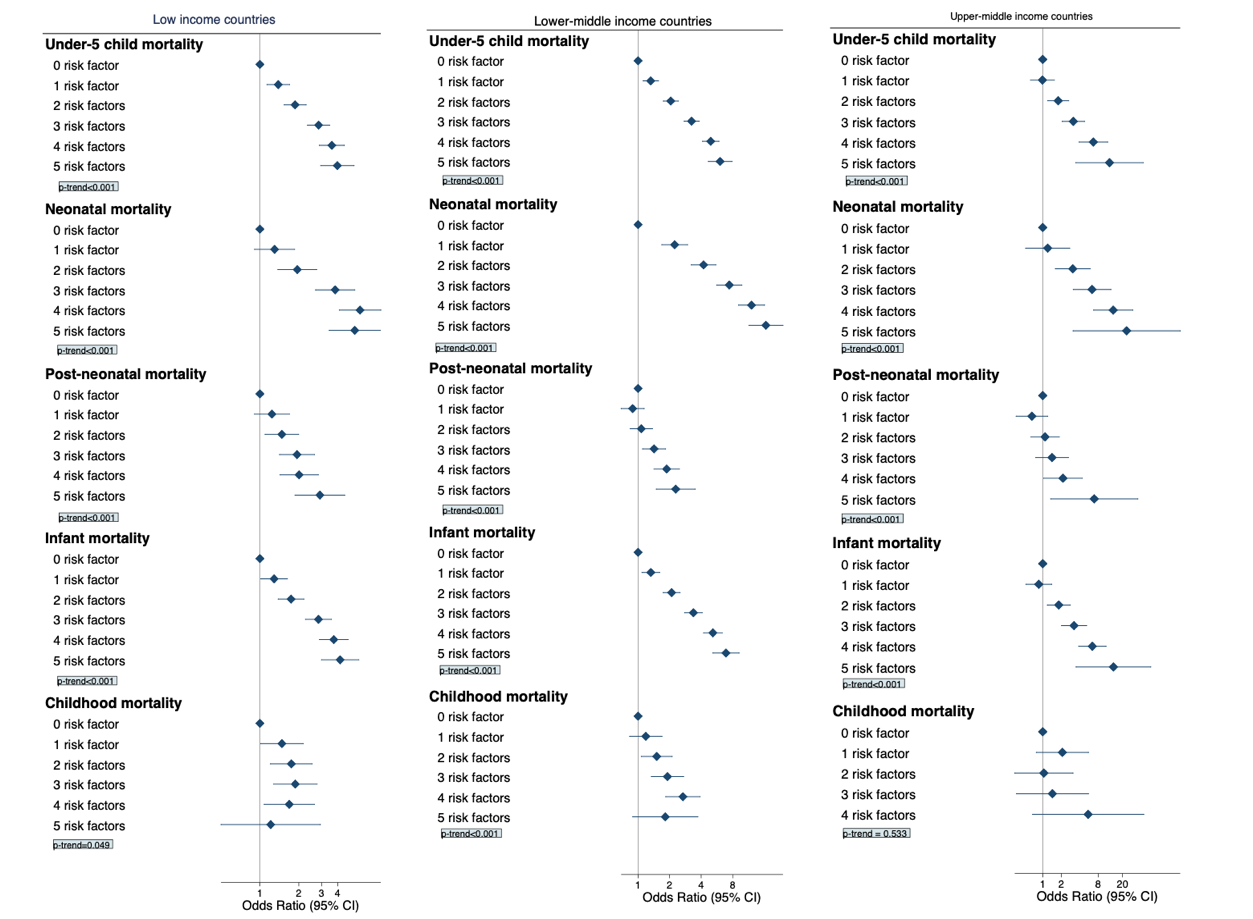


* The concurrence of multiple risk factors was computed by counting the presence of the top 5 risk factors (i.e., unsatisfied family planning, short birth interval (<18 months), small birth size, never breastfed or delayed breastfeeding, and low maternal education (no schooling)) for each individual.

* Neonatal mortality – deaths from 0 to 28 days, post-neonatal mortality – deaths from 29 to 364 days, infant mortality – deaths from 0 to 364 days, childhood mortality – deaths from 365 days to 59 months.

**Appendix Figure 8. Association between concurrence of multiple leading risk factors with under-5 mortality; odds ratio (ORs) and 95% confidence interval (CI), missing value as separate categories.**

* Child age and country-fixed effects were adjusted in this model

* Short birth interval – birth interval <18m; Low maternal education – mothers with no education; ANC less than 4 times – antenatal care visit less than 4 times

**Appendix Figure 9. Association between concurrence of multiple leading risk factors with under-5 mortality, neonatal mortality, post-neonatal mortality, infant mortality, and childhood mortality; odds ratio (ORs) and 95% confidence interval (CI), stratified by armed conflict events**


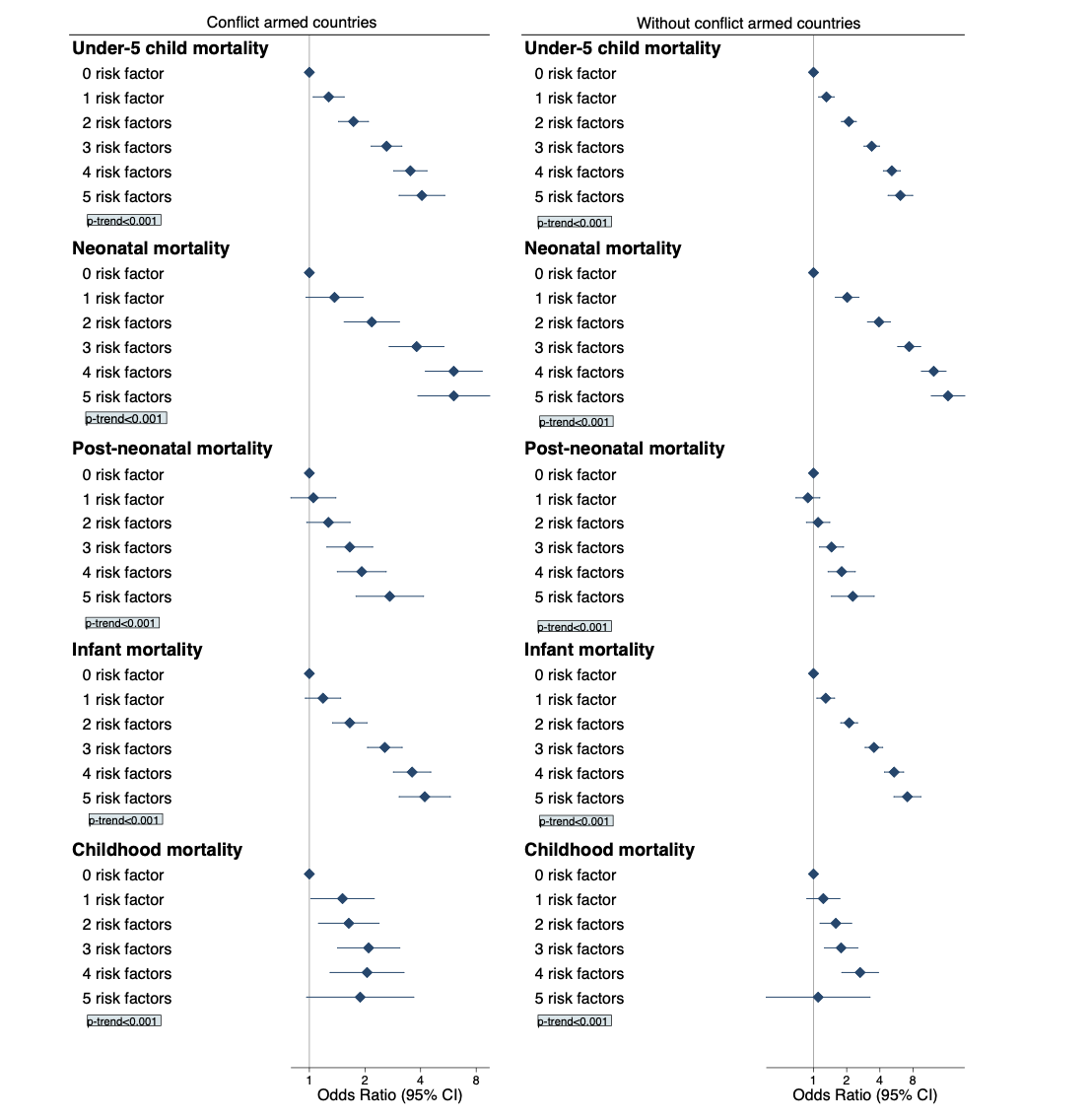


* The concurrence of multiple risk factors was computed by counting the presence of the top 5 risk factors (i.e., short birth interval (<18 months), small birth size, never breastfed or delayed breastfeeding, low maternal education (no schooling), and unsatisfied family planning) for each individual.

* Neonatal mortality – deaths from 0 to 28 days, post-neonatal mortality – deaths from 29 to 364 days, infant mortality – deaths from 0 to 364 days, childhood mortality – deaths from 365 days to 59 months.

*****Countries with armed conflicted events at the time of survey: Afghanistan, Burundi, Chad, Colombia, Congo Democratic Republic, Coted' Ivoire, Egypt, Ethiopia, Gabon, Ghana, Haiti, Kenya, Lesotho, Mali, Mozambique, Myanmar, Namibia, Nigeria, Pakistan, Tanzania, Togo, Uganda, Yemen, and Zimbabwe.

**Appendix Figure 10. Association between concurrence of 12 significant risk factors with under-5 mortality, neonatal mortality, post-neonatal mortality, infant mortality, and childhood mortality; odds ratio (ORs) and 95% confidence interval (CI)**

**^*^**The concurrence of multiple risk factors was computed by counting the presence of the all 13 risk factors (i.e., unsatisfied family planning, short birth interval (<18 months), small birth size, never breastfed or delayed breastfeeding, low maternal education (no schooling), indoor air pollution, maternal age at birth <19 y/o, maternal height <145cm, ANC less than 4 times, birth order- after 6^th^, formerly or never married, no tetanus injection during pregnancy) for each individual.

* Neonatal mortality – deaths from 0 to 28 days, post-neonatal mortality – deaths from 29 to 364 days, infant mortality – deaths from 0 to 364 days, childhood mortality – deaths from 365 days to 59 months.

**Appendix Figure 11. Association between concurrence of multiple leading risk factors with under-5 mortality, neonatal mortality, post-neonatal mortality, infant mortality, and childhood mortality; odds ratio (ORs) and 95% confidence interval (CI), 33 countries with observation on all risk factors of interest**

* The concurrence of multiple risk factors was computed by counting the presence of the top 5 risk factors (i.e., unsatisfied family planning, short birth interval (<18 months), small birth size, never breastfed or delayed breastfeeding, low maternal education (no schooling)) for each individual.

*Neonatal mortality – deaths from 0 to 28 days, post-neonatal mortality – deaths from 29 to 364 days, infant mortality – deaths from 0 to 364 days, childhood mortality – deaths from 365 days to 59 months.

*33 countries with observation on all risk factors of interest: Benin, Burkina Faso, Burundi, Cameroon, Comoros, Congo, Congo Democratic Republic, Coted' Ivoire, Gabon, Gambia , Ghana, Guinea, Haiti, India , Kenya, Liberia, Madagascar, Malawi, Mali, Mauritania, Mozambique, Myanmar, Namibia , Niger, Nigeria, Pakistan, Rwanda , Sierra Leone, Tanzania, Timor Leste, Togo, Uganda, Zimbabwe.

**Appendix Figure 12. Association between concurrence of multiple leading risk factors with under-5 mortality, neonatal mortality, post-neonatal mortality, infant mortality, and childhood mortality; odds ratio (ORs) and 95% confidence interval (CI), No-MI dataset**

* The concurrence of multiple risk factors was computed by counting the presence of the top 5 risk factors (i.e., unsatisfied family planning, short birth interval (<18 months), small birth size, never breastfed or delayed breastfeeding, low maternal education (no schooling)) for each individual.

* Neonatal mortality – deaths from 0 to 28 days, post-neonatal mortality – deaths from 29 to 364 days, infant mortality – deaths from 0 to 364 days, childhood mortality – deaths from 365 days to 59 months.

**Appendix Figure 13. Association between concurrence of multiple leading risk factors with under-5 mortality, neonatal mortality, post-neonatal mortality, infant mortality, and childhood mortality; odds ratio (ORs) and 95% confidence interval (CI), with adjustment of year-fixed effects.**

* The concurrence of multiple risk factors was computed by counting the presence of the top 5 risk factors (i.e., unsatisfied family planning, short birth interval (<18 months), small birth size, never breastfed or delayed breastfeeding, low maternal education (no schooling)) for each individual.

* Neonatal mortality – deaths from 0 to 28 days, post-neonatal mortality – deaths from 29 to 364 days, infant mortality – deaths from 0 to 364 days, childhood mortality – deaths from 365 days to 59 months.

**Figure 14. Association between concurrence of multiple leading risk factors with under-5 mortality, neonatal mortality, post-neonatal mortality, infant mortality, and childhood mortality; odds ratio (ORs) and 95% confidence interval (CI), including all children without application of any exclusion criteria (N= 851,255)**

* The concurrence of multiple risk factors was computed by counting the presence of the top 5 risk factors (i.e., unsatisfied family planning, short birth interval (<18 months), small birth size, never breastfed or delayed breastfeeding, low maternal education (no schooling)) for each individual.

* Neonatal mortality – deaths from 0 to 28 days, post-neonatal mortality – deaths from 29 to 364 days, infant mortality – deaths from 0 to 364 days, childhood mortality – deaths from 365 days to 59 months.
